# Assessing the effects of HMGCR, LPL, and PCSK9 inhibition on sleep apnea: Mendelian randomization analysis of drug targets

**DOI:** 10.1097/MD.0000000000040194

**Published:** 2024-10-25

**Authors:** Wei Tan, Xiujuan Deng, Xiaoning Tan, Guangbo Tan

**Affiliations:** a Graduate School, Hunan University of Chinese Medicine, Changsha, China; b Department of Pulmonology, Affiliated Hospital of Hunan Academy of Traditional Chinese Medicine, Changsha, China; c Department of Oncology, Affiliated Hospital of Hunan Academy of Traditional Chinese Medicine, Changsha, China

**Keywords:** lipid, lipid-lowering drug, PCSK9 inhibitors, sleep apnea syndrome, statins

## Abstract

To investigate the use of lipid-lowering drugs and abnormal serum lipid levels in patients at risk of sleep apnea syndrome. Three types of Mendelian randomization (MR) analyses were used. First, a 2-sample Mendelian randomization (TSMR) analysis was used to investigate the association between sleep apnea syndrome risk and serum lipid levels. Multivariate Mendelian randomization (MVMR) analysis was subsequently used to investigate the effects of confounding variables on SAS incidence of sleep apnea syndrome. Finally, drug-target Mendelian randomization (DMR) analysis was used to analyze the association between lipid-lowering drug use and sleep apnea syndrome risk. According to the TSMR analysis, the serum HDL-C concentration was negatively correlated with sleep apnea syndrome (OR = 0.904; 95% CI = 0.845–0.967; *P* = .003). Serum TG levels were positively correlated with sleep apnea syndrome (OR = 1.081; 95% CI = 1.003–1.163; *P* = .039). The association between serum HDL-C levels and sleep apnea syndrome in patients with MVMR was consistent with the results in patients with TSMR (OR = 0.731; 95% CI = 0.500–1.071; *P* = 3.94E−05). According to our DMR analysis, HMGCR and PCSK9, which act by lowering serum LDL-C levels, were inversely associated with the risk of sleep apnea syndrome (OR = 0.627; 95% CI = 0.511–0.767; *P *= 6.30E−06) (OR = 0.775; 95% CI = 0.677–0.888; *P *= .0002). LPL, that lowered serum TG levels, was positively associated with the risk of sleep apnea syndrome (OR = 1.193; 95% CI = 1.101–1.294; *P* = 1.77E−05). Our analysis suggested that high serum HDL-C levels may reduce the risk of sleep apnea syndrome. Low serum TG levels have a protective effect against sleep apnea syndrome. The DMR results suggested that the use of HMGCR lipid-lowering drugs (such as statins) and PCSK9 inhibitors has a protective effect against sleep apnea syndrome. However, LPL-based lipid-lowering drugs may increase the risk of sleep apnea syndrome.

## 1. Introduction

Sleep apnea syndrome is the most common form of breathing disorder, and close to 1 billion people worldwide may suffer from sleep apnea syndrome.^[1]^ Over the past 30 years, researchers have found that apnea during sleep is a serious health problem.^[2]^ Despite increasing awareness of sleep apnea syndrome, it still poses a significant threat to public health resources, the economy, and overall well-being.^[3]^ The progressive development of sleep apnea syndrome can lead to a series of respiratory system diseases, cardiovascular diseases, cerebrovascular diseases, and, in some cases, sudden death at night.^[4]^ Further study of the risk factors for this disease is important to ensure patient health.

In 2018, a cross-sectional study of 8592 patients from the European Sleep Apnea Database investigating the association between sleep apnea syndrome and lipid concentrations showed that the severity of sleep apnea syndrome was independently associated with cholesterol and TG concentrations.^[5]^ Studies have shown that abnormal serum HDL-C levels are associated with sleep apnea syndrome, and dyslipidemia is more pronounced in patients with severe obstructive apnea.^[6]^ At present, there are still large differences in the use of lipid-lowering drugs for sleep apnea syndrome. A multicenter randomized controlled trial showed no significant improvement in endothelial function or atherosclerosis in patients with obstructive sleep apnea.^[7]^ In addition, studies have shown that obstructive sleep apnea is closely related to PCSK9, and patients with sleep apnea who take statins have elevated PCSK9.^[8]^ A PCSK9 inhibitor lowers serum LDL-C concentrations.^[9]^ However, research on the risk of sleep apnea associated with lipid-lowering drugs is lacking, and further investigations on the effects of statins and PCSK9 inhibitors on this disease are needed.

PCSK9 inhibitors are compounds that inhibit PCSK9,^[10]^ currently serving as a novel class of lipid-lowering drugs.^[11]^ PCSK9 inhibitors are monoclonal antibodies primarily used for the treatment of hypercholesterolemia.^[12]^ PCSK9 inhibitors inhibit the binding of PCSK9 to LDL receptors,^[13]^ thereby preventing the PCSK9-mediated degradation of LDL receptors,^[14]^ increasing the number of LDL receptors on the surface of hepatocytes,^[15]^ and subsequently promoting the clearance of LDL-C to reduce LDL-C levels in the blood.^[16]^ HMGCR is a key enzyme in cholesterol synthesis process.^[17]^ It exists in the endoplasmic reticulum of the liver,^[18]^ intestine, and other tissues and is a glycoprotein composed of 887 amino acid residues. HMGCR is a rate-limiting enzyme in cholesterol synthesis^[19]^ and its activity directly affects the rate of cholesterol synthesis, thereby influencing cholesterol levels in the body. Owing to its crucial role in cholesterol synthesis, HMGCR has become an important target for cholesterol-lowering drugs.^[20]^ Currently, the primary HMGCR inhibitors used clinically are statins^[21]^ such as mevastatin, pravastatin, and lovastatin. These drugs possess structural fragments similar to HMG-CoA,^[22]^ enabling them to competitively inhibit the reduction of HMG-CoA to mevalonate, thereby reducing the amount of cholesterol synthesized.

Clinical studies have limitations, such as confounders, survival bias, and reverse causality, which can cause errors or contradictions in observational results.^[23]^ However, these problems can be avoided using Mendelian randomization (MR).^[24]^ The TSMR was used to study the correlation between the exposure and outcome factors. Its advantage is that it can eliminate the interference from confounding factors. Mendelian randomization of drug targets (DMR) can provide important information for drugs,^[25]^ such as lipid-lowering drugs, including predicting efficacy and revealing target-mediated adverse reactions.^[26]^ In this study, we used the TSMR to analyze the causal relationship between sleep apnea syndrome and serum lipid levels. In the DMR analysis, we evaluated the impact of various lipid-lowering drugs on SAS incidence of sleep apnea syndrome.

## 2. Materials and methods

### 2.1. Study design

**Figure 1.**
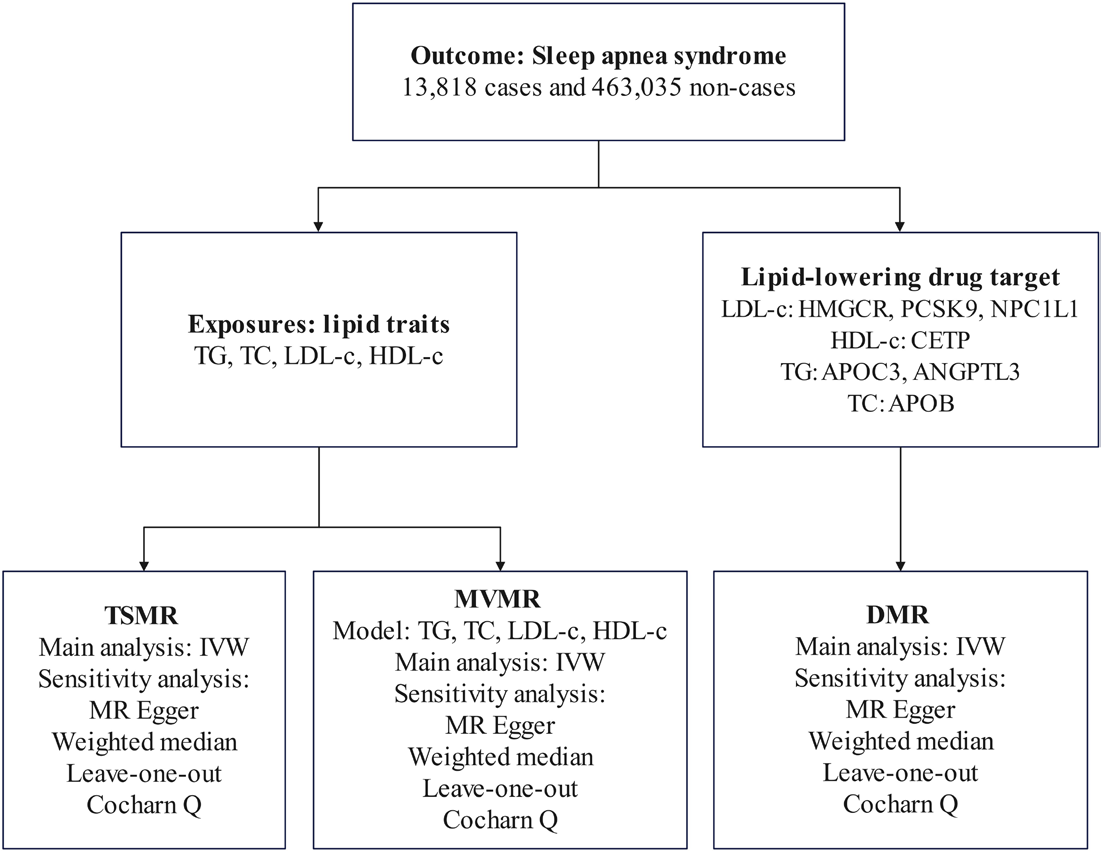
Design scheme of this study.

A research flowchart is shown in Figure 1. Three MR analyses were then performed. First, the effects of different lipids (TG, TC, LDL-C, and HDL-C^[27]^) on the risk of sleep apnea syndrome were evaluated using TSMR analysis. Next, we used MVMR analysis to explore the effects of meaningful variables in the TSMR on sleep apnea syndrome. Finally, we performed DMR analysis to assess the effects of the lipid-lowering drugs HMGCR, PCSK9, NPC1L1, CETP, APOB, LPL, APOC3, and ANGPTL3 on the risk of sleep apnea syndrome.^[28]^

### 2.2. GWAS data sources

Statistical data for sleep apnea syndrome were obtained from the 476,853 European Descent GWAS meta-analysis (https://gwas.mrcieu.ac.uk/). From this meta-analysis, we identified 13,818 patients with sleep apnea syndrome and 463,035 healthy controls. All blood lipid data were obtained from a free IEU database (https://gwas.mrcieu.ac.uk/). After several screenings, we obtained a highly representative dataset. All data included in this study are presented in Table S1, Supplemental Digital Content, http://links.lww.com/MD/N781. Ethical requirements such as patient informed consent were waived in accordance with the original study ethics approval from the GWAS.

### 2.3. Instrumental variable selection

In the TSMR analysis, first, we obtained single nucleotide polymorphisms (SNPs) for sleep apnea syndrome from the publicly available database of the European Bioinformatics Institute. We subsequently screened the SNPs as follows: genome-wide significance threshold *P* < 5 × 10^−8[29]^; linkage imbalance *R*^2^ value < 0.001^[30]^; distance between adjacent SNPs <10 Mb; palindromic instrumental variables (IVs) excluding intermediate allelic frequencies; and threshold *F*-statistic > 10.^[31]^ Finally, we identified that SNPs were strongly associated with sleep apnea syndrome. We used the selected SNPs as IVs for TSMR analysis. MVMR analysis is based on TSMR to further analyze the effects of multiple exposures on sleep apnea syndrome. This method has the advantage of avoiding bias due to confusion.^[32]^ In DMR analysis, the association between eqtl and GWAS-based IVs was confirmed by DMR analysis using eqtl in the 100 kb range on both sides of the coding gene. The screening criteria for IVs were the same as those previously described.

### 2.4. MR analyses

Inverse variance weighting (IVW) is the primary analysis method for TSMR.^[33]^ Horizontal pleiotropy was determined using the MR-Egger method, whereas heterogeneity and directed pleiotropy were estimated using Cochran Q statistic and the MR-Egger test.^[33]^ The MR-PRESSO method removes all “outliers” from the original SNP dataset to improve the reliability of the results.^[34]^ We used these methods to analyze the association between lipid levels and risk of sleep apnea syndrome. In the MVMR analysis, we used IVW as the main analysis method. Methods for TSMR analysis were used to determine horizontal pleiotropy, heterogeneity, and directed pleiotropy and to remove all “outliers.”^[26]^ In DMR analysis, IVW was used as the primary method to analyze the association between various lipid-lowering drugs and the risk of sleep apnea syndrome. Heterogeneity was detected using Cochran *Q* test (*P* < .05). Horizontal pleiotropy was evaluated by MR- the MR-MR-Egger regression and MR-PRESSO analyses (*P* > .05). We used these methods to analyze the causal relationship between lipid-lowering drugs and sleep apnea syndrome.

## 3. Results

### 3.1. Instrumental variable

In the TSMR analysis, we used SNPs that were closely associated with sleep apnea syndrome and screened them, as described in Section 2.3. We also used the harmonize function to adjust for the exposure and outcome results (Tables S2–S5, Supplemental Digital Content, http://links.lww.com/MD/N781).

### 3.2. Causal effect of lipids on sleep apnea syndrome

**Figure 2.**
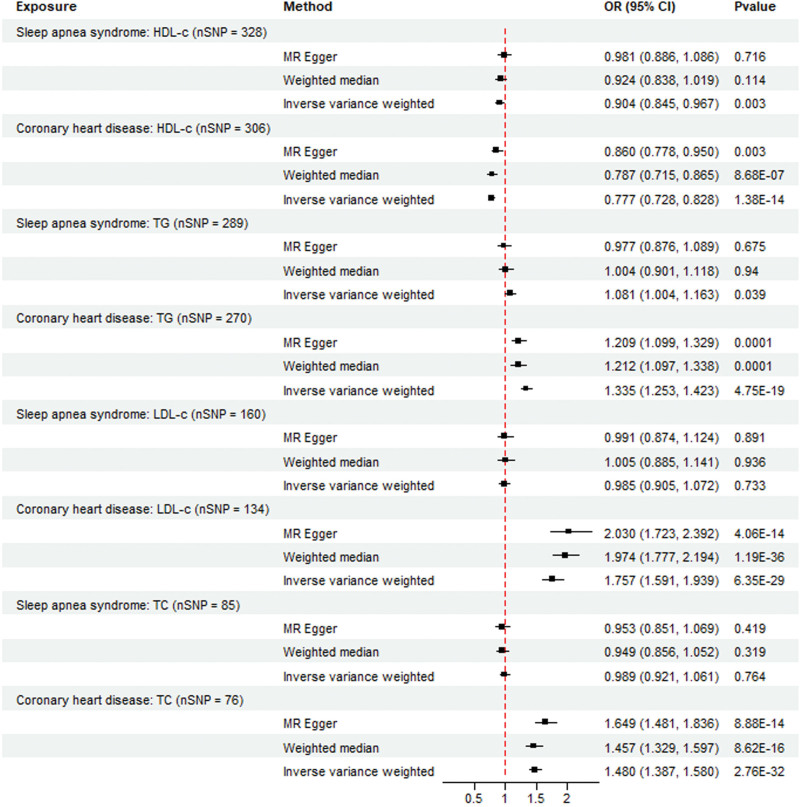
The results of the TSMR analysis of the associations between LDL-C, HDL-C, TG, and TC and sleep apnea syndrome risk were obtained using the IVW, MR-Egger, and weighted median methods. The lipid levels mentioned above were validated using CHD as a positive control. number of SNPs.

The number of SNPs with a causal relationship between lipid markers and sleep apnea syndrome was as follows: TG, 289; TC, 85; LDL-C, 160; and HDL-C, 328 (Fig. 2; Tables S2–S5, Supplemental Digital Content, http://links.lww.com/MD/N781). In the TSMR analysis, we used the IVW analysis, MR, and weighted median method as auxiliary methods. Prior to the IVW analysis, we used MR-PRESSO analysis, which was designed to eliminate outliers and enhance the stability of the results. According to TSMR analysis, serum HDL-C levels were negatively correlated with sleep apnea syndrome (OR = 0.904; 95% CI = 0.845–0.967; *P* = .003) (Figs. S1–S3, Supplemental Digital Content, http://links.lww.com/MD/N780). Serum TG levels were positively correlated with sleep apnea syndrome (OR = 1.081; 95% CI = 1.003–1.163; *P* = .039) (Figs. S4–S6, Supplemental Digital Content, http://links.lww.com/MD/N780). However, the results of this study did not reveal any association between serum TC or LDL-C levels and sleep apnea. We selected CHD patients as positive controls to verify the presence of these lipid-associated SNPs. The P-values for LDL-C, HDL-C, TG, and TC in the serum were 6.35E−29, 1.38E−14, 4.75E−19, and 2.76E−32, respectively (Figs. S40–S51, Supplemental Digital Content, http://links.lww.com/MD/N780; Table S9, Supplemental Digital Content, http://links.lww.com/MD/N781).

### 3.3. Investigation of the effect of blood lipids on sleep apnea syndrome using the MVMR

**Figure 3.**
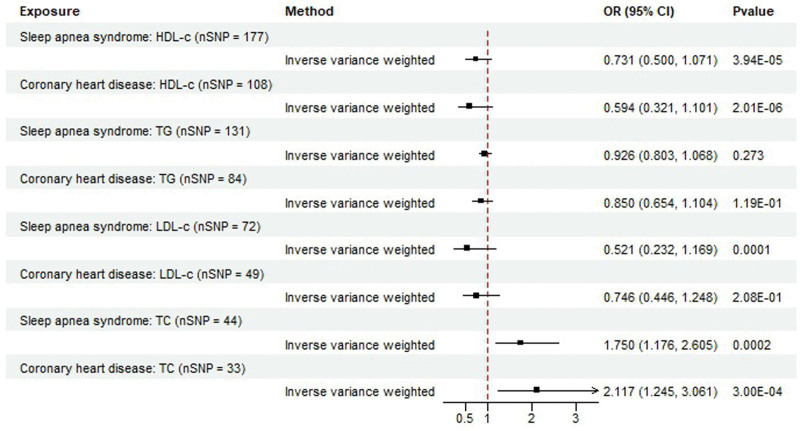
Results of MVMR analysis.

Based on the results of the TSMR analysis, when MVMR analysis was used to investigate the effects of serum LDL-C, HDL-C, TG, and TC levels on sleep apnea syndrome, a high serum HDL-C level was shown to have a protective effect on sleep apnea syndrome (OR = 0.731; 95% CI = 0.500–1.071; *P *= 3.94E−05) (Fig. 3; Table S10, Supplemental Digital Content, http://links.lww.com/MD/N781). However, the results of our MVMR analysis differed from those of the TSMR analysis. When we examined the effects of serum HDL-C and TG levels on patient outcomes, we found that the serum TC and LDL-C levels were associated with sleep apnea syndrome. MVMR analysis revealed that serum total cholesterol (TC) levels were positively correlated with sleep apnea syndrome (OR = 1.750; 95% CI = 1.176–2.605; *P* = .0002), and serum LDL-C levels were negatively correlated with sleep apnea syndrome (OR = 0.521; 95% CI = 0.232–1.169; *P* = .0001) (Fig. 3; Table S10, Supplemental Digital Content, http://links.lww.com/MD/N781). Similarly, we selected patients with CHD as positive controls to verify the presence of lipid-associated SNPs. The *P*-value for serum HDL-C concentration was 2.01E−06 (Table S10, Supplemental Digital Content, http://links.lww.com/MD/N781).

### 3.4. Causal effect of lipid-lowering drugs on the risk of sleep apnea syndrome

**Figure 4.**
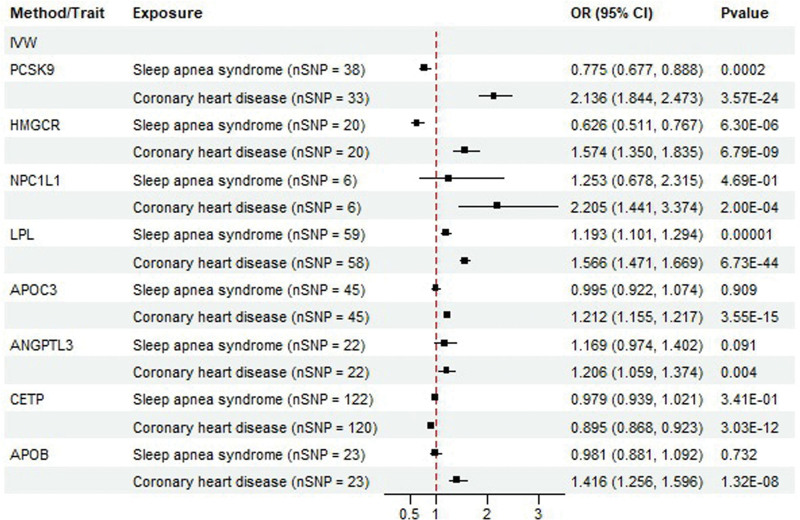
DMR analysis of the 8 drug targets. DMR validation analysis of the above drug targets and CHD incidence. Number of SNPs.

DMR analysis revealed that HMGCR and PCSK9, which lower serum LDL-C levels, were negatively associated with the risk of sleep apnea syndrome (OR = 0.627; 95% CI = 0.511–0.767; *P *= 6.30E−06) (OR = 0.775; 95% CI = 0.677–0.888; *P* = .0002) (Fig. 4; Figs. S7–S12, Supplemental Digital Content, http://links.lww.com/MD/N780; and Table S6, Supplemental Digital Content, http://links.lww.com/MD/N781). LPL, that lowers serum TG levels, was positively associated with the risk of sleep apnea syndrome (OR = 1.193; 95% CI = 1.101–1.294; *P *= 1.77E−05) (Figs. S13–S15, Supplemental Digital Content, http://links.lww.com/MD/N780). However, our results did not reveal any association between the lipid-lowering drugs NPC1L1, CETP, APOB, APOC3, or ANGPTL3, and the risk of sleep apnea syndrome (Table S7, Supplemental Digital Content, http://links.lww.com/MD/N781). As in the TSMR analysis, we selected patients with CHD as positive controls to verify the presence of lipid-lowering drug-associated SNPs. The *P*-values for HMGCR, PCSK9, NPC1L1, CETP, APOB, LPL, APOC3, and ANGPTL3 were 6.78E−09, 3.57E−24, 0.0002, 3.03E−12, 1.32E−08, 6.97E−44, 3.55E−15, and .004, respectively (Figs. S16–S39, Supplemental Digital Content, http://links.lww.com/MD/N780; Table S8, Supplemental Digital Content, http://links.lww.com/MD/N781).

## 4. Discussion

Sleep apnea syndrome is the most common form of sleep disorder and is very common in morbidly obese,^[35]^ elderly, and asthmatic patients.^[36]^ The pathological mechanisms of sleep apnea syndrome include hypoxia, dyslipidemia, and hypertension, which are associated with respiratory and circulatory risk factors.^[37]^ Dyslipidemia is associated with sleep apnea syndrome, mainly obstructive sleep apnea.^[38]^ Drugs that lower blood lipid levels have been clinically proven useful in the treatment of respiratory and cardiovascular diseases. However, its effect on sleep apnea syndrome at the genetic level remains unclear. Therefore, we performed a TSMR analysis to explore the causal relationship between changes in the serum levels of various lipid factors and the risk of sleep apnea syndrome. Next, we used MVMR analysis to explore the effects of meaningful variables in the TSMR on sleep apnea syndrome. Finally, we performed a DMR analysis to assess the effect of lipid-lowering drugs on the risk of sleep apnea syndrome.

Our TSMR analysis suggests that high serum HDL-C levels reduce the risk of sleep apnea syndrome. Low serum TG levels have a protective effect against sleep apnea syndrome. Moreover, when we used MVMR analysis to explore the effects of serum LDL-C, HDL-C, TG, and TC levels on sleep apnea syndrome, we showed that high serum HDL-C levels had a protective effect on sleep apnea syndrome. However, the results of this study did not reveal any correlation between serum HDL-C or TC levels and sleep apnea. A cross-sectional study showed a positive association between sleep apnea syndrome and serum TG levels.^[39]^ Additionally, a clinical observational study of 215 patients with sleep apnea syndrome suggested that an increase in LDL-C/HDL-C was positively correlated with the risk of sleep apnea syndrome, and a significant decrease was observed after continuous positive airway pressure.^[40]^ These findings are consistent with the results of the MR analysis.

Previous studies have found that patients with sleep apnea have significantly increased PCSK9 levels and larger plasma low-density lipoprotein diameters after statin use.^[8]^ According to Sanja Jelic, statins may be 1 way to improve the health of people with sleep apnea.^[41]^ The study found that statins, but not CPAP (continuous positive airway pressure), protected blood vessels from dangerous inflammatory changes that occur in patients with sleep apnea. In addition to traditional statins, several novel drugs have shown potential for the treatment of OSA and dyslipidemia. For example, tizepatide, as a double agonist, can act on both GIP and GLP-1 receptors simultaneously, with hypoglycemic, weight loss, and lipid improvement effects. In a Phase 3 clinical trial of tilpotide for sleep apnea, the primary endpoint of the apnea hypopnea index decreased by 22.6 to 24.4 compared with placebo, and body weight decreased by 16.8% to 17.8%.^[42]^ A multicenter randomized controlled trial showed that while atorvastatin regulates lipid levels, it does not have any therapeutic effect on endothelial function in patients with sleep apnea syndrome.^[7]^ However, statins have demonstrated advantages in the treatment of obstructive pulmonary disease, statins have demonstrated their advantages.^[43]^ There is a lack of research in this area examining the relationship between lipid-lowering drugs and sleep apnea syndrome. Therefore, we performed a DMR analysis to investigate the association between lipid-lowering drugs and the risk of sleep apnea syndrome. Our DMR results suggest that HMGCR lipid-lowering drugs such as statins and PCSK9 inhibitors have a protective effect against sleep apnea syndrome. However, LPL-based lipid-lowering drugs may increase the risk of sleep apnea syndrome. Taken together, these findings and the results of the TSMR analysis suggest that the protective effect of HMGCR lipid-lowering drugs and PCSK9 inhibitors on sleep apnea syndrome may not be related to a reduction in the serum LDL-C concentration. However, the underlying mechanism requires further investigation.

Our study had the following advantages. First, this study is the first to use GWAS data to establish a causal relationship between serum TG and HDL-C levels and sleep apnea syndrome. Serum TG and HDL-C levels can be used as experimental parameters to predict the risk of sleep apnea syndrome. Second, we concluded from the genetic data analysis that the overuse of lipid-lowering drugs, represented by LPL, increases the risk of sle6ep apnea syndrome. Third, we verified the accuracy of the MR data using the following methods: we calculated the *F*-statistic to remove weakly correlated IVs; we used patients with CHD as a positive control group to verify the reliability of SNPs; we performed MVMR analysis to avoid bias due to confounding; we used the IVW method to verify the accuracy of the drug-target MR analysis results; and we used genetic tools instead of drug exposure for drug target MR analysis to minimize confounding bias and reverse causation.

However, our study had several limitations. The sleep apnea syndrome database used in this study is limited to individuals from continental Europe. Therefore, we cannot easily use these data to explain the risk of sleep apnea syndrome in other countries or populations in other continents.

## 5. Conclusion

Our analysis suggests that high serum HDL-C and low serum TG levels reduce the risk of sleep apnea syndrome. The use of HMGCR lipid-lowering drugs (such as statins) and PCSK9 inhibitors has a protective effect against sleep apnea. However, lipid-lowering drugs may increase the risk of sleep apnea syndrome. However, the mechanism of action of HMGCR lipid-lowering drugs and PCSK9 inhibitors in sleep apnea syndrome requires further investigation. The development of new lipid-lowering drugs for the treatment of sleep apnea syndrome is a trend in the future.

## Acknowledgments

The authors would like to thank all members of their research group for their helpful comments.

## Author contributions

**Conceptualization:** Xiujuan Deng.

**Data curation:** Wei Tan.

**Formal analysis:** Wei Tan, Xiaoning Tan.

**Funding acquisition:** Guangbo Tan.

**Investigation:** Wei Tan.

**Methodology:** Wei Tan.

**Software:** Wei Tan.

**Writing – original draft:** Wei Tan.

**Writing – review & editing:** Wei Tan, Xiaoning Tan, Guangbo Tan.

## Supplementary Material


