# Supplementary material for: Assessing the effects of HMGCR, LPL, and PCSK9 inhibition on sleep apnea: Mendelian randomization analysis of drug targets
Source: Medicine (Baltimore). 2024 Oct 25;103(43):e40194. doi: 10.1097/MD.0000000000040194 (PMC11520985; doi:10.1097/MD.0000000000040194)
Supplement: Supplementary file 2 [file medi-103-e40194-s002.pdf]

Supplementary Material

Supplementary Figures

HDL-c & Sleep apnea syndrome Scatter plot

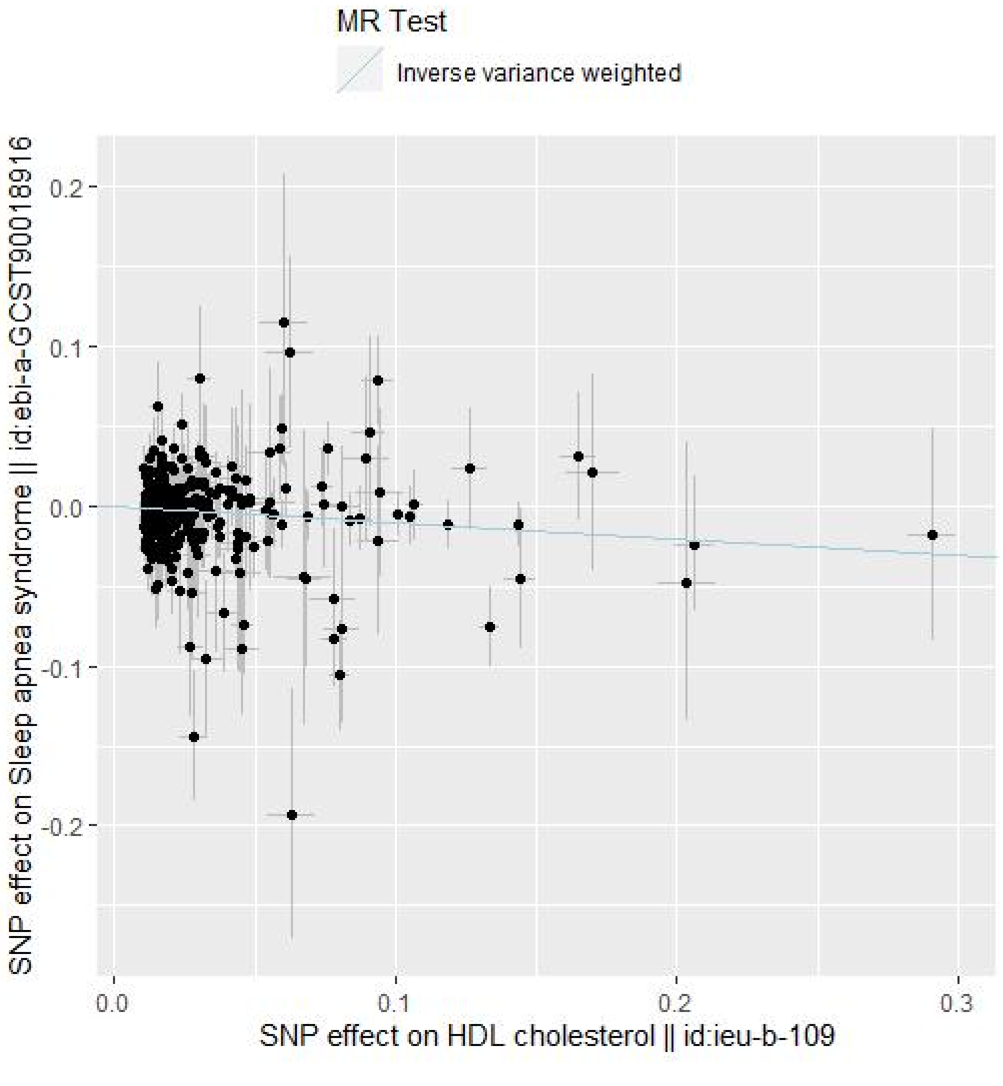

Supplemental Figure 1. Scatter plot of between serum HDL-c and Sleep apnea syndrome TSMR analysis. HDL-c, high-density lipoprotein cholesterol; TSMR, two-sample Mendelian randomization.

HDL-c & Sleep apnea syndrome leave-one-out analysis

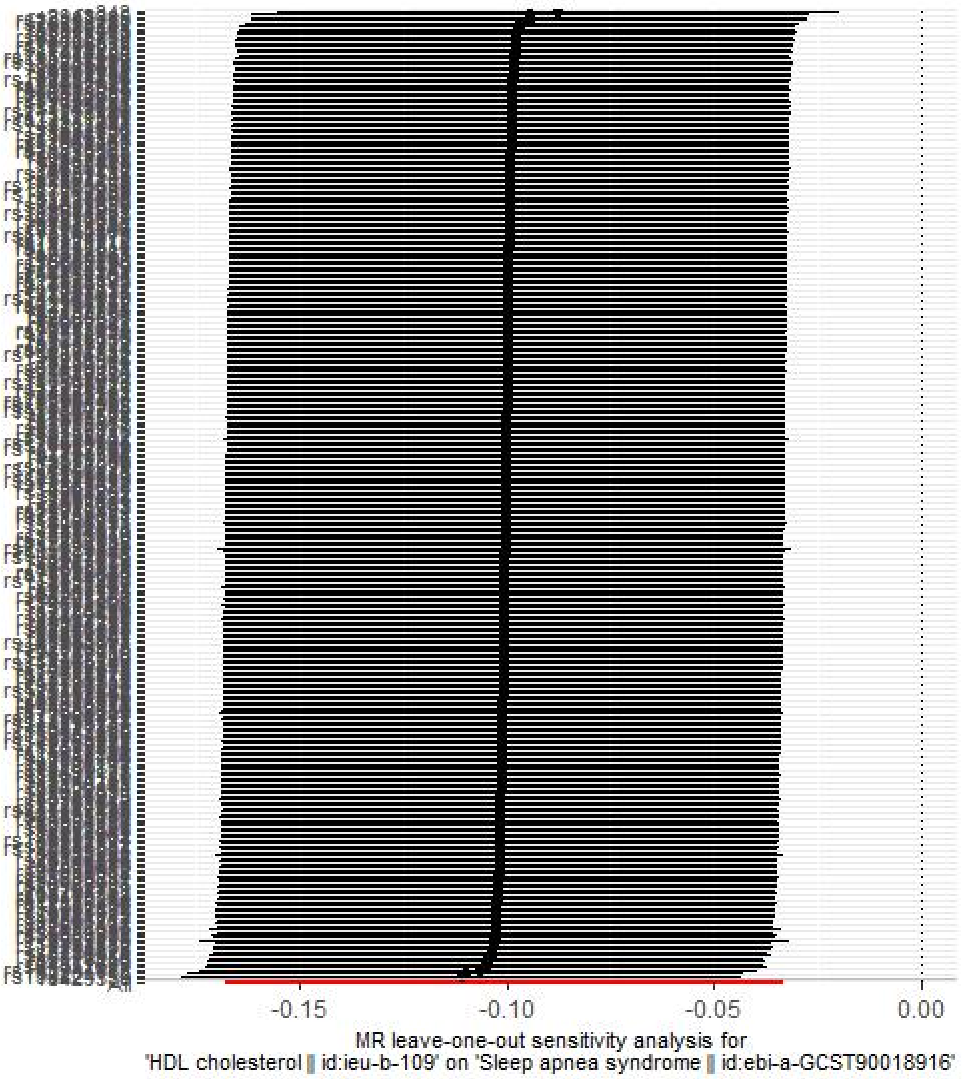

Supplemental Figure 2. Serum HDL-c and Sleep apnea syndrome TSMR leave-one-out analysis. HDL-c, high-density lipoprotein cholesterol; TSMR, two-sample Mendelian randomization

HDL-c & Sleep apnea syndrome funnel plot

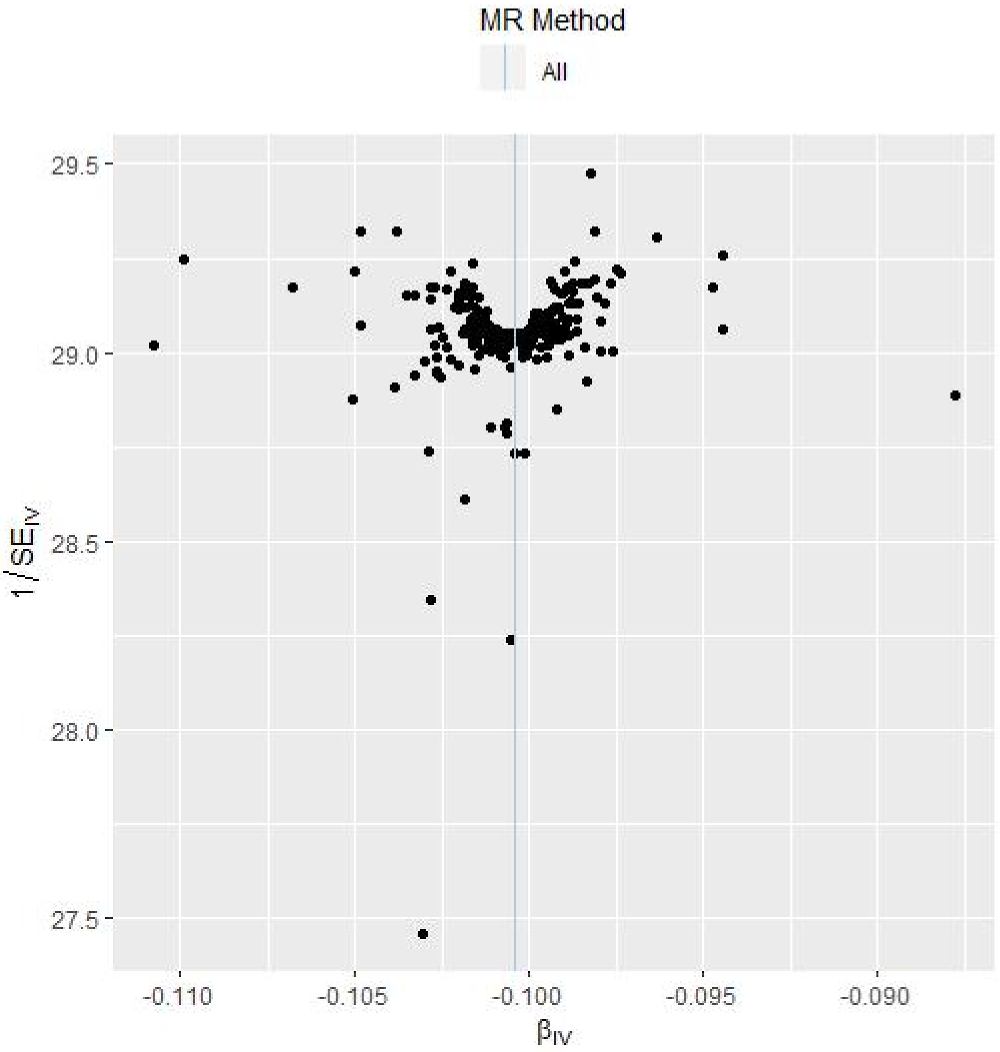

Supplemental Figure 3. Funnel plot of between serum HDL-c and Sleep apnea syndrome TSMR analysis. HDL-c, high-density lipoprotein cholesterol; TSMR, two-sample Mendelian randomization.

TG & Sleep apnea syndrome Scatter plot

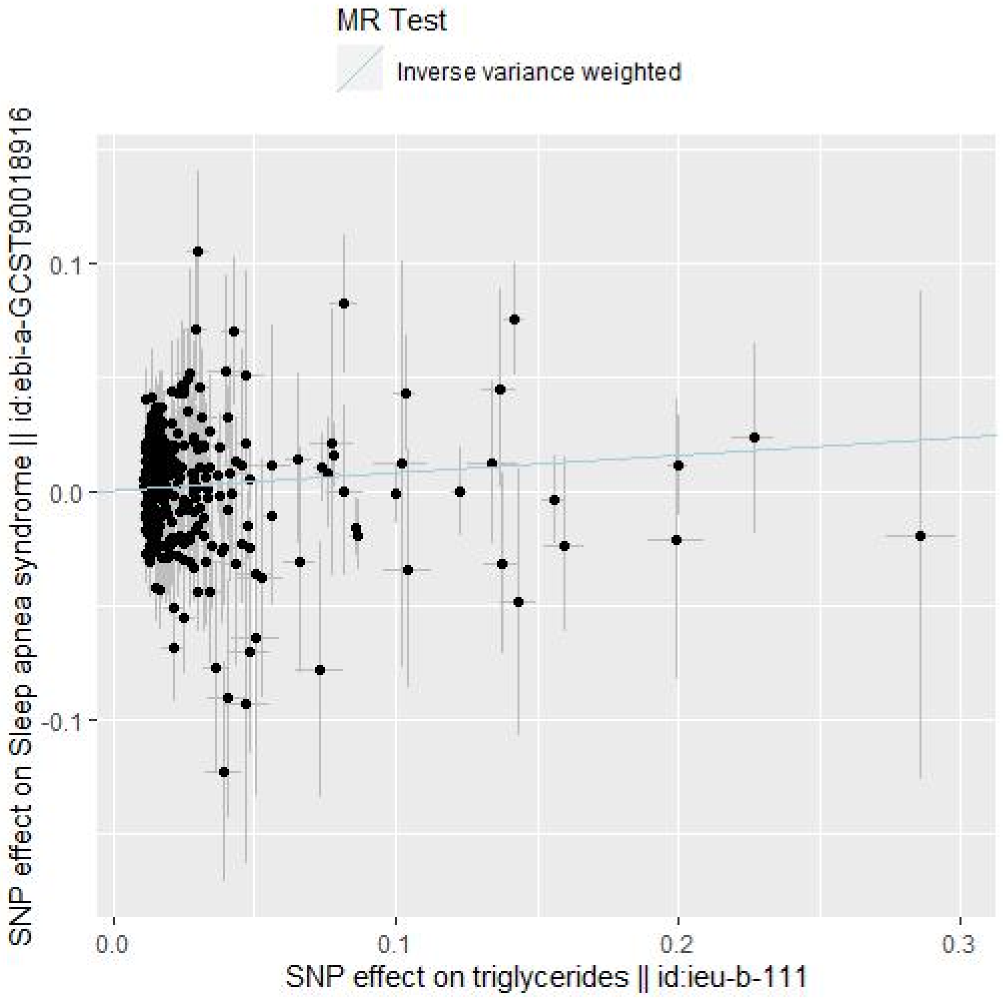

Supplemental Figure 4. Scatter plot of between serum TG and Sleep apnea syndrome TSMR. TG, total triglycerides; TSMR, two-sample Mendelian randomization

TG &Sleep apnea syndrome leave-one-out analysis

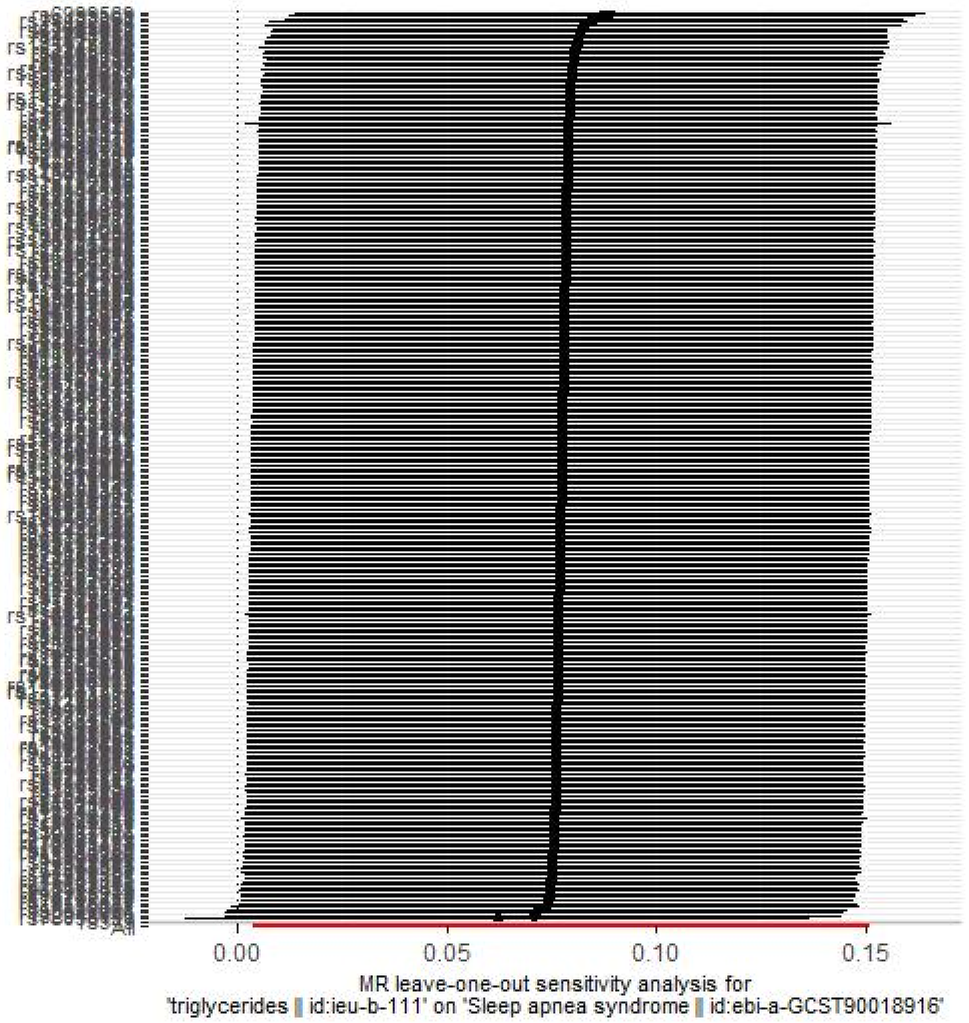

Supplemental Figure 5. Serum TG andSleep apnea syndrome TSMR leave-one-out analysis. TG, total triglycerides; TSMR, two-sample Mendelian randomization.

TG &Sleep apnea syndrome funnel plot

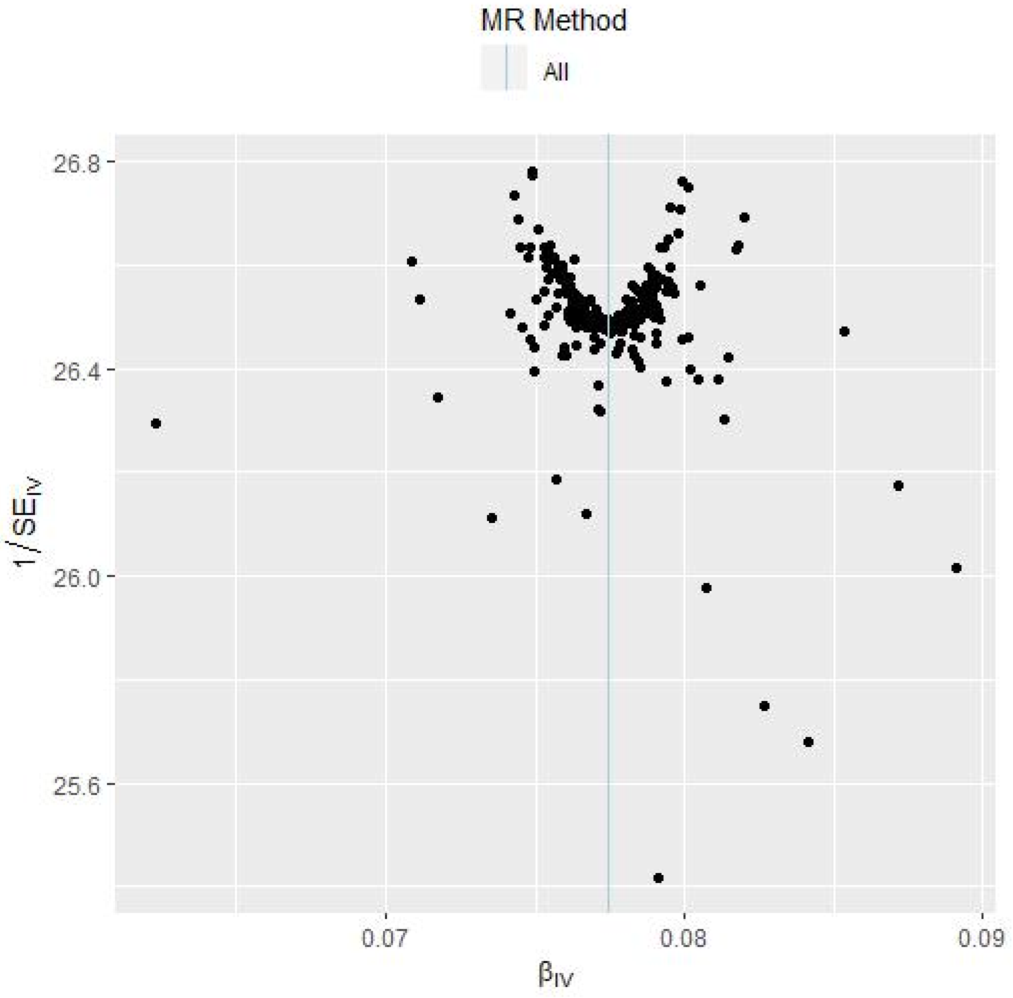

Supplemental Figure 6. Funnel plot of between serum TG andSleep apnea syndrome TSMR. TC, total triglycerides; TSMR, two-sample Mendelian randomization.

PCSK9 &Sleep apnea syndrome Scatter plot

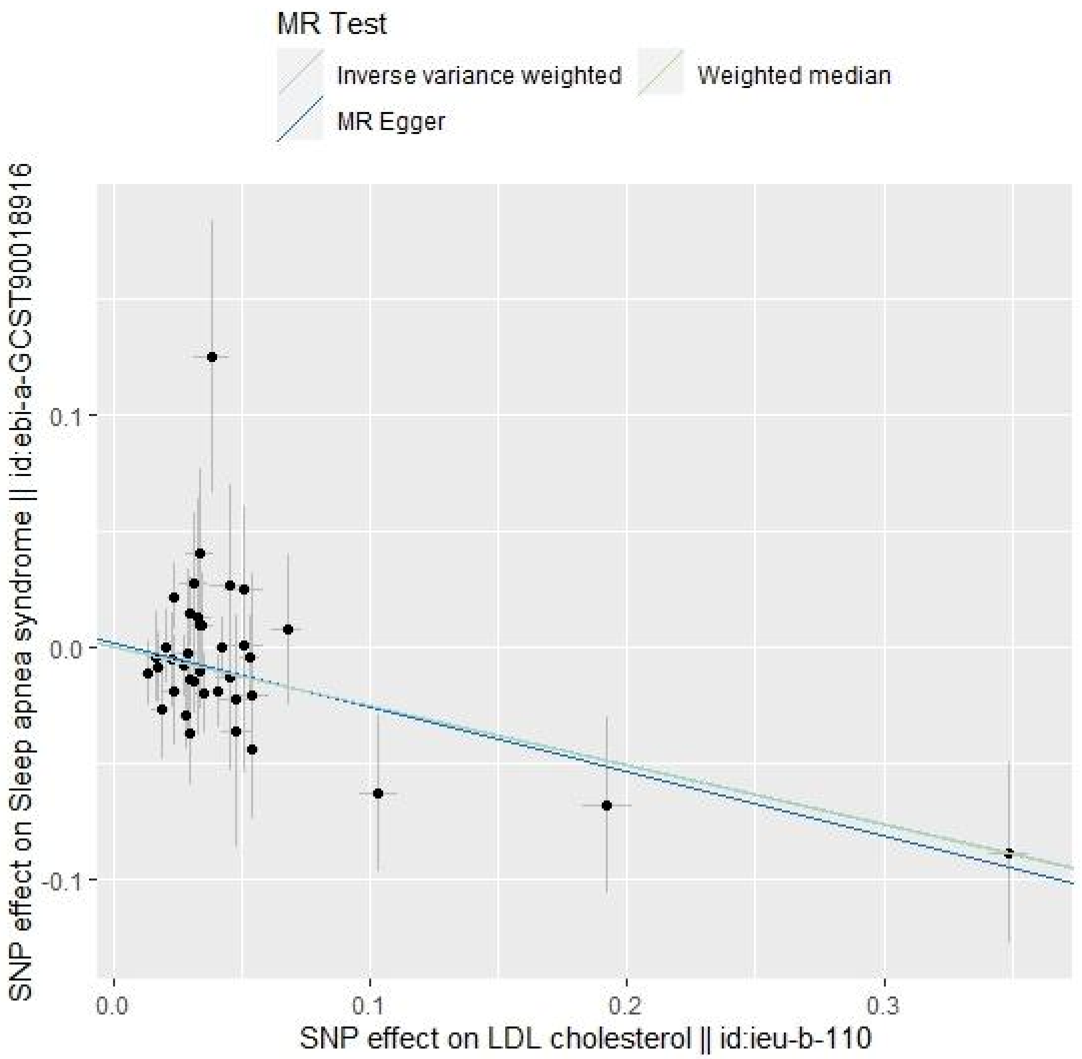

Supplemental Figure 7. Scatter plot of betweenPCSK9 and Sleep apnea syndrome DMR. DMR, drug target Mendelian randomization.

PCSK9 &Sleep apnea syndrome leave-one-out analysis

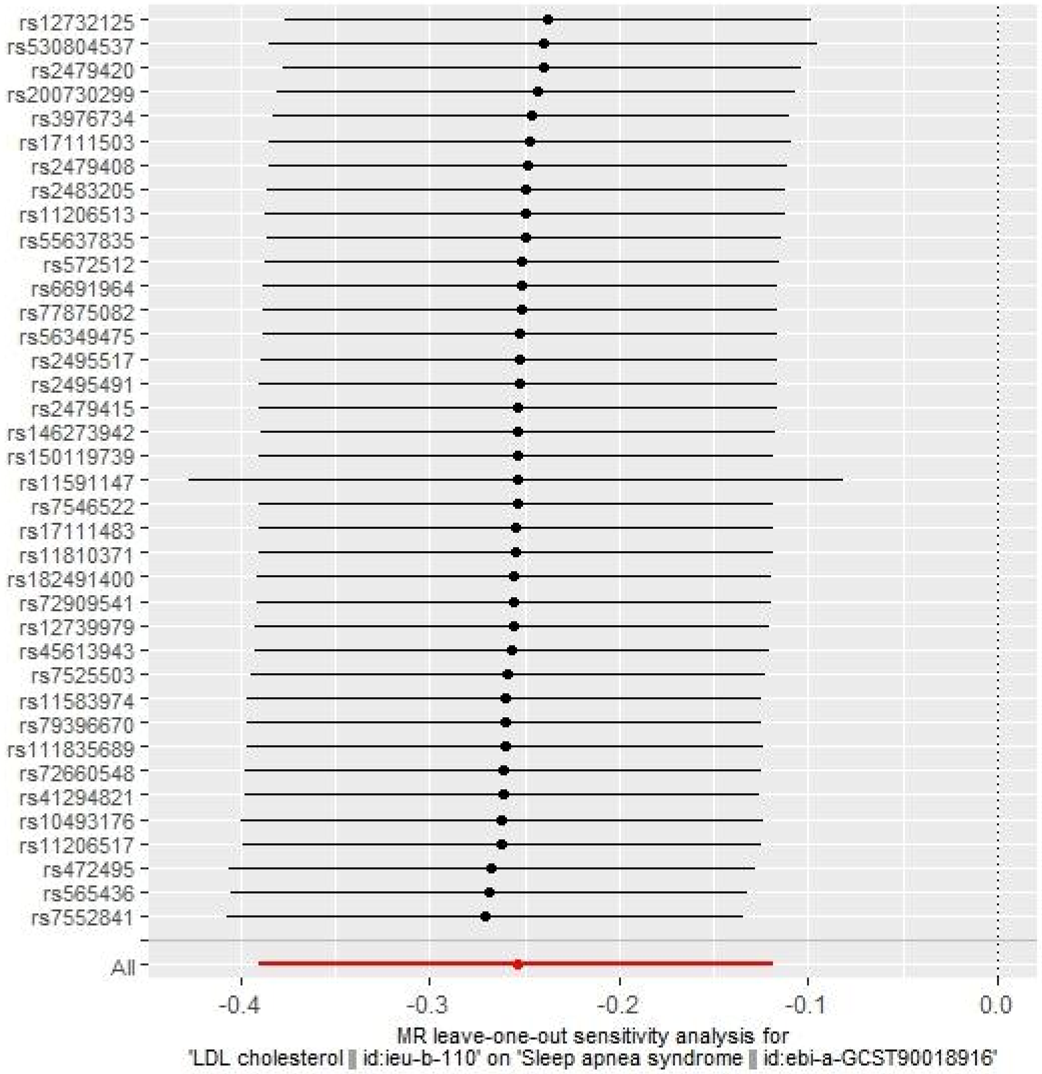

Supplemental Figure 8.PCSK9 and Sleep apnea syndrome DMR leave-one-out analysis. DMR, drug target Mendelian randomization.

PCSK9 &Sleep apnea syndrome funnel plot

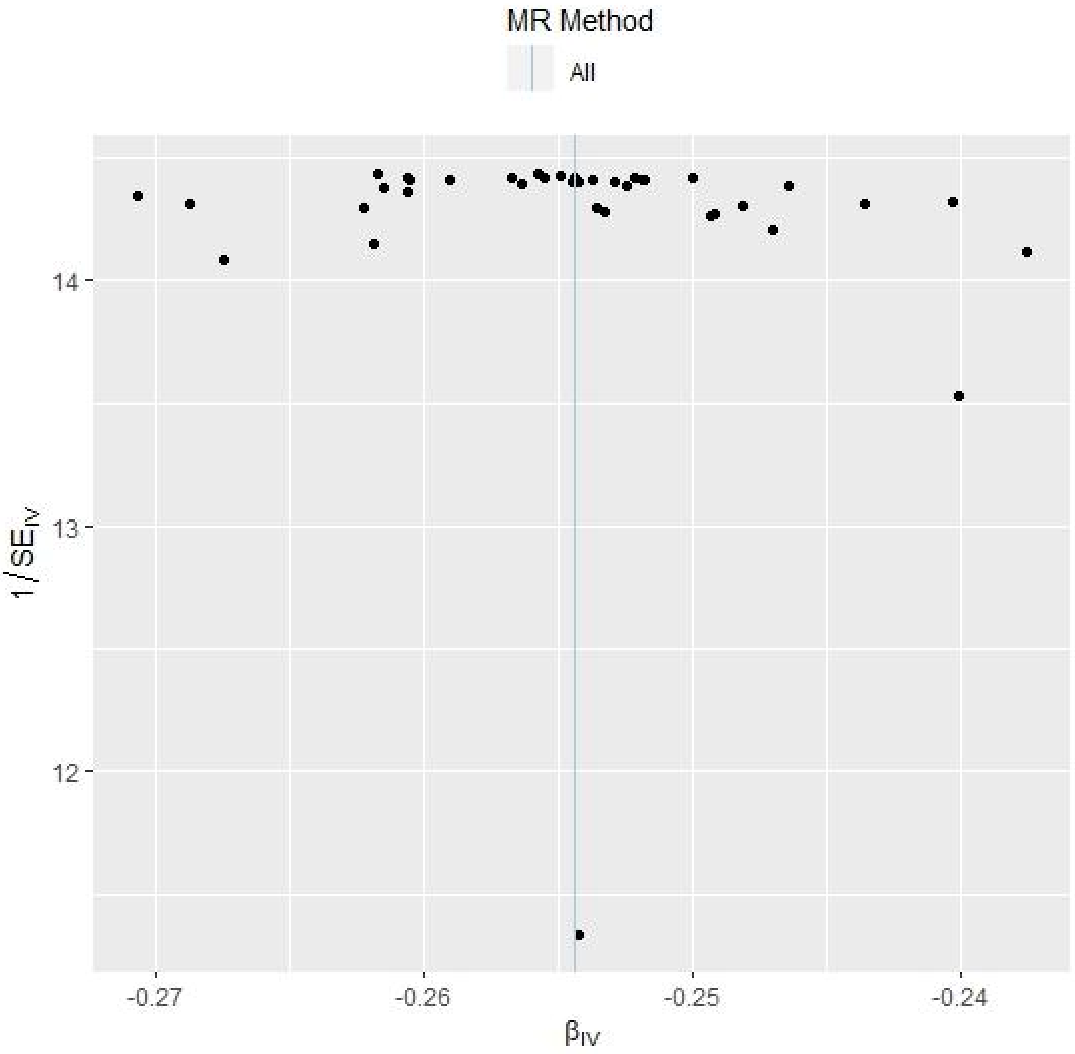

Supplemental Figure 9. Funnel plot of between PCK9 andSleep apnea syndrome DMR. DMR, drug target Mendelian randomization.

HMGCR &Sleep apnea syndrome Scatter plot

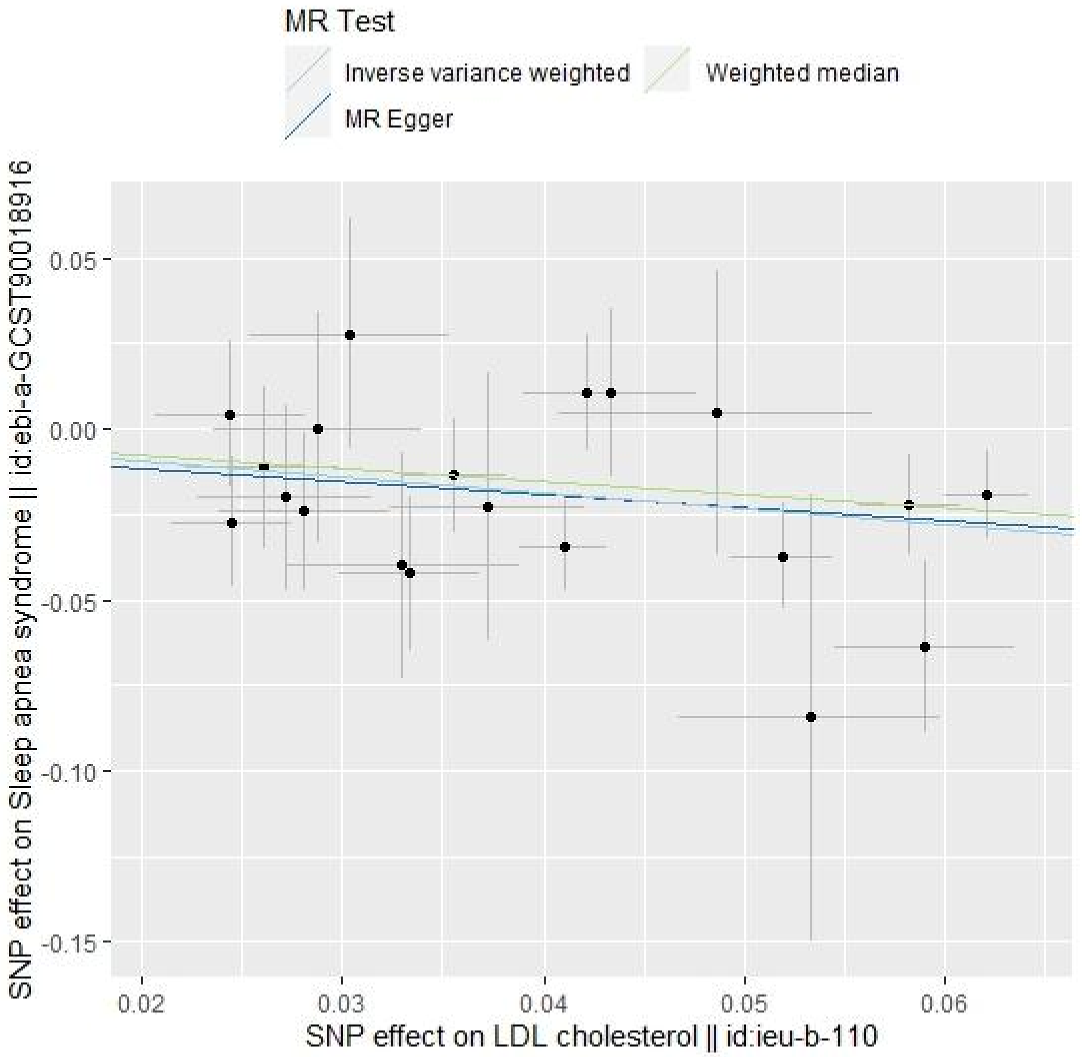

Supplemental Figure 10. Scatter plot of between HMGCR and Sleep apnea syndrome DMR. DMR, drug target Mendelian randomization.

HMGCR &Sleep apnea syndrome leave-one-out analysis

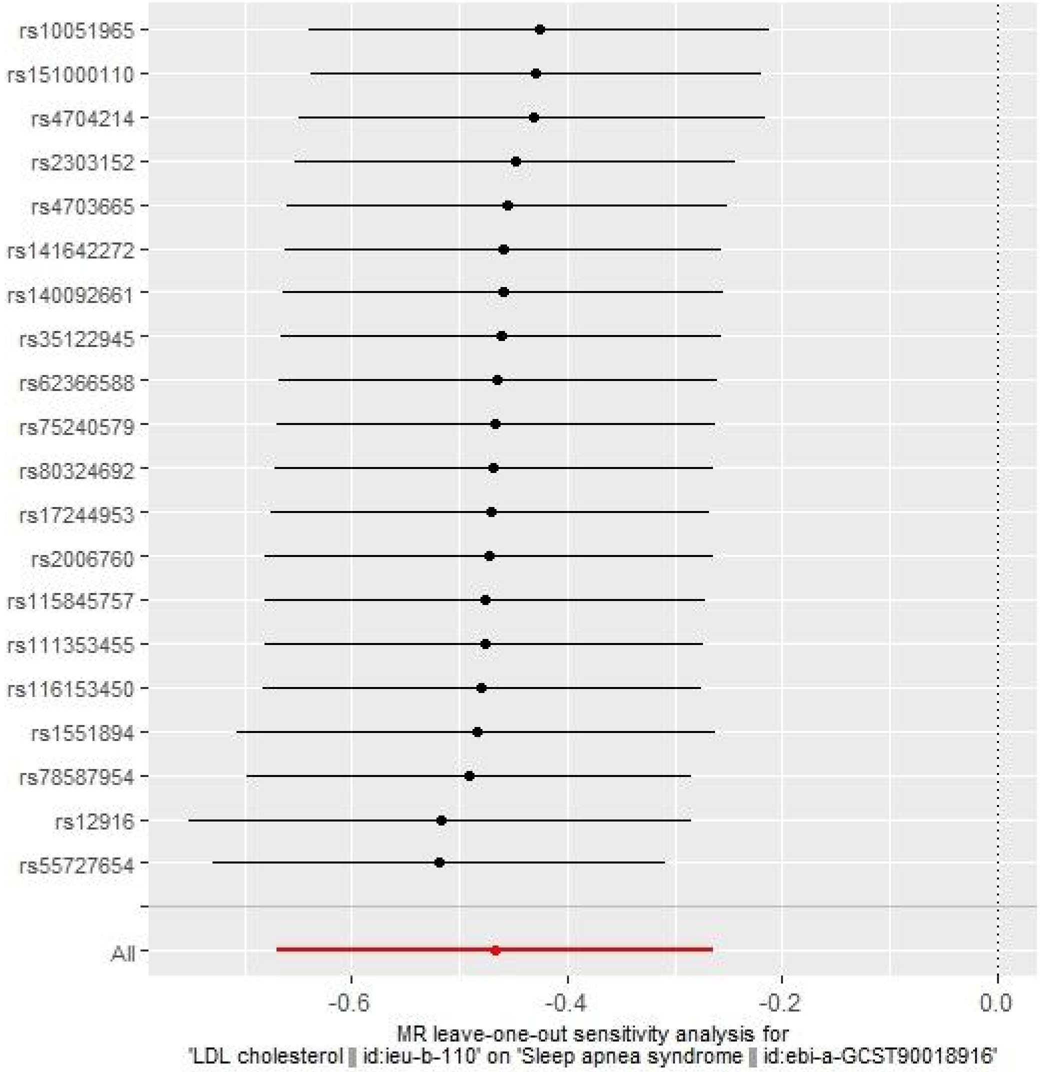

Supplemental Figure 11. HMGCR and Sleep apnea syndrome DMR leave-one-out analysis. DMR, drug target Mendelian randomization.

HMGCR & Sleep apnea syndrome funnel plot

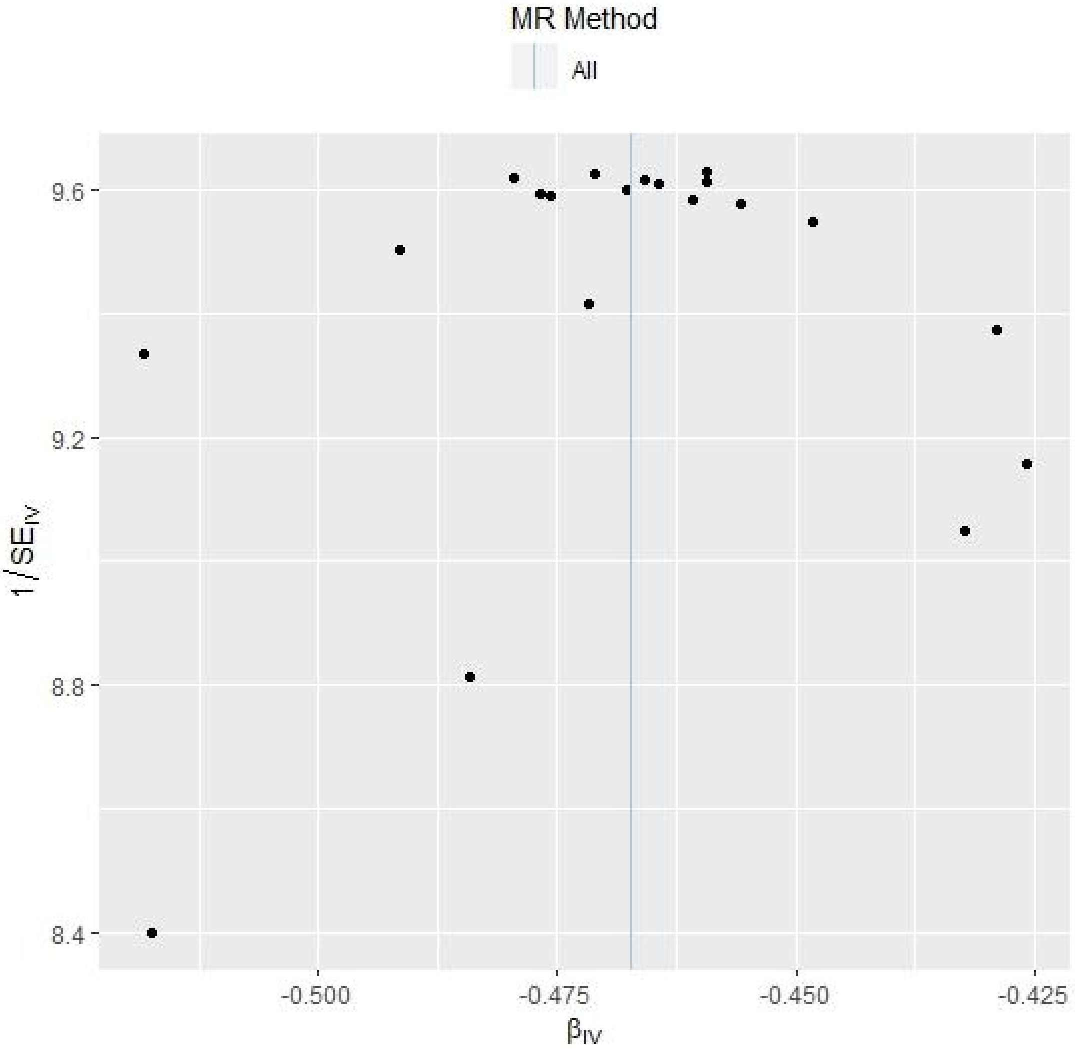

Supplemental Figure 12. Funnel plot of between HMGCR andSleep apnea syndrome DMR. DMR, drug target Mendelian randomizatio

LPL & Sleep apnea syndrome Scatter plot

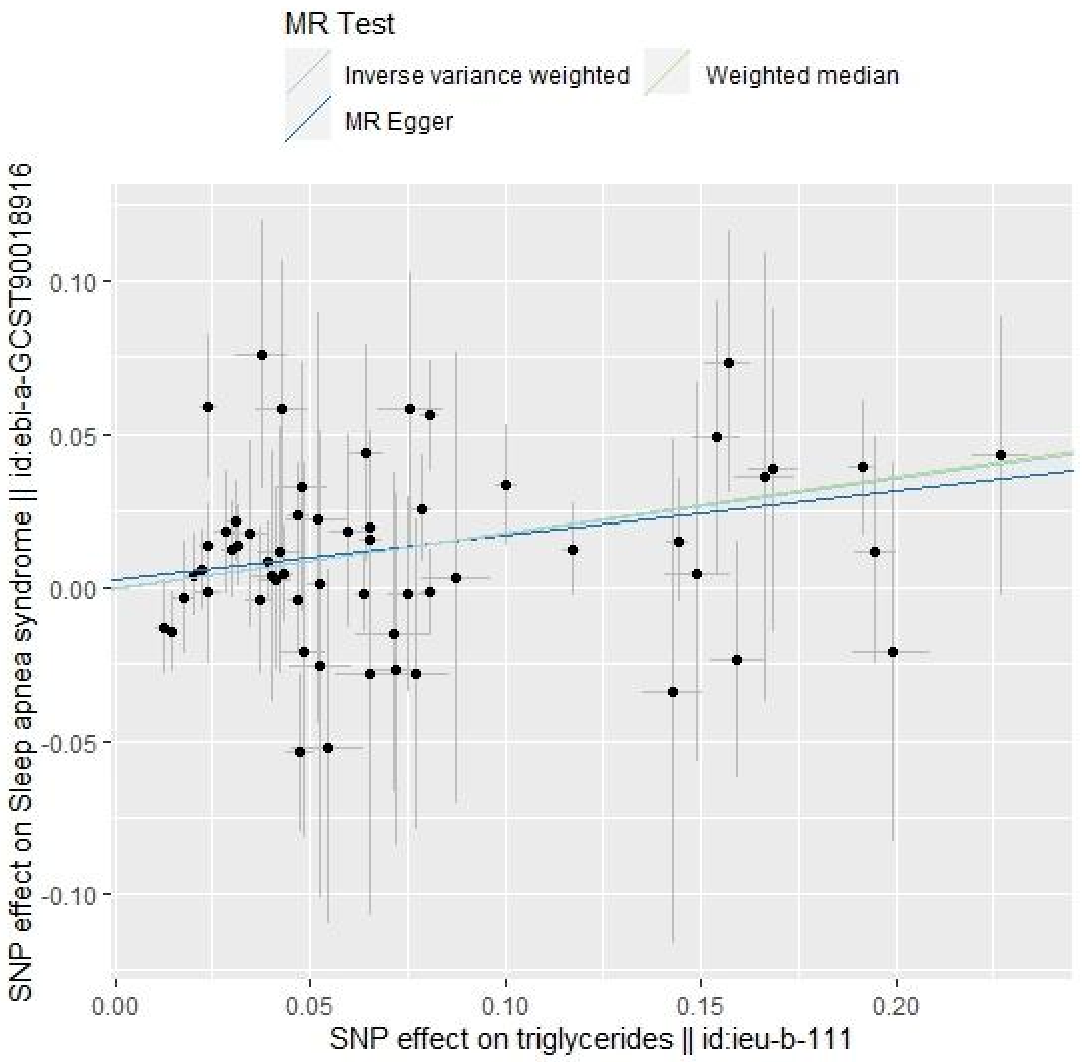

Supplemental Figure 13. Scatter plot of between LPL and Sleep apnea syndrome DMR. DMR, drug target Mendelian randomization

LPL & Sleep apnea syndrome leave-one-out analysis

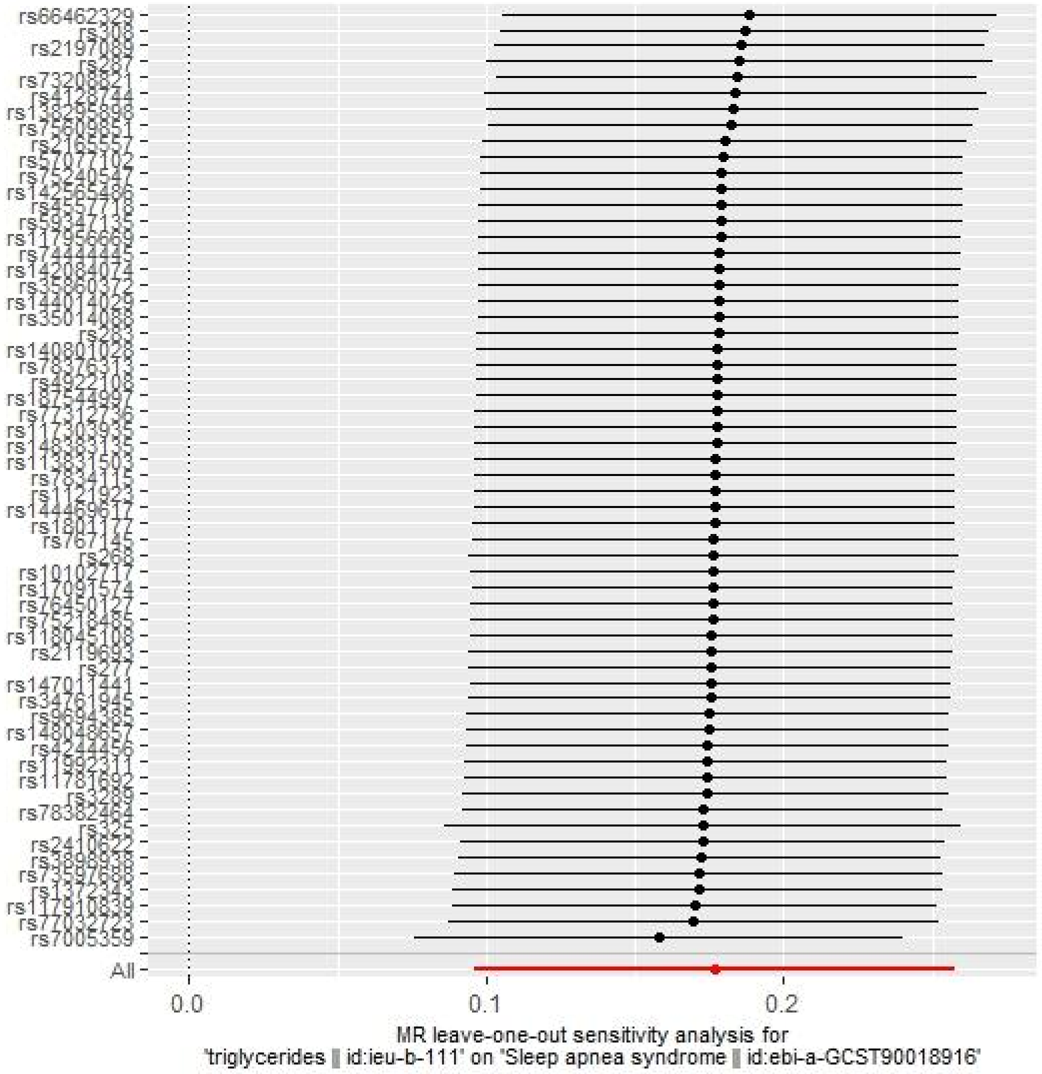

Supplemental Figure 14. LPL and Sleep apnea syndrome DMR leave-one-out analysis. DMR, drug target Mendelian randomization.

LPL & Sleep apnea syndrome funnel plot

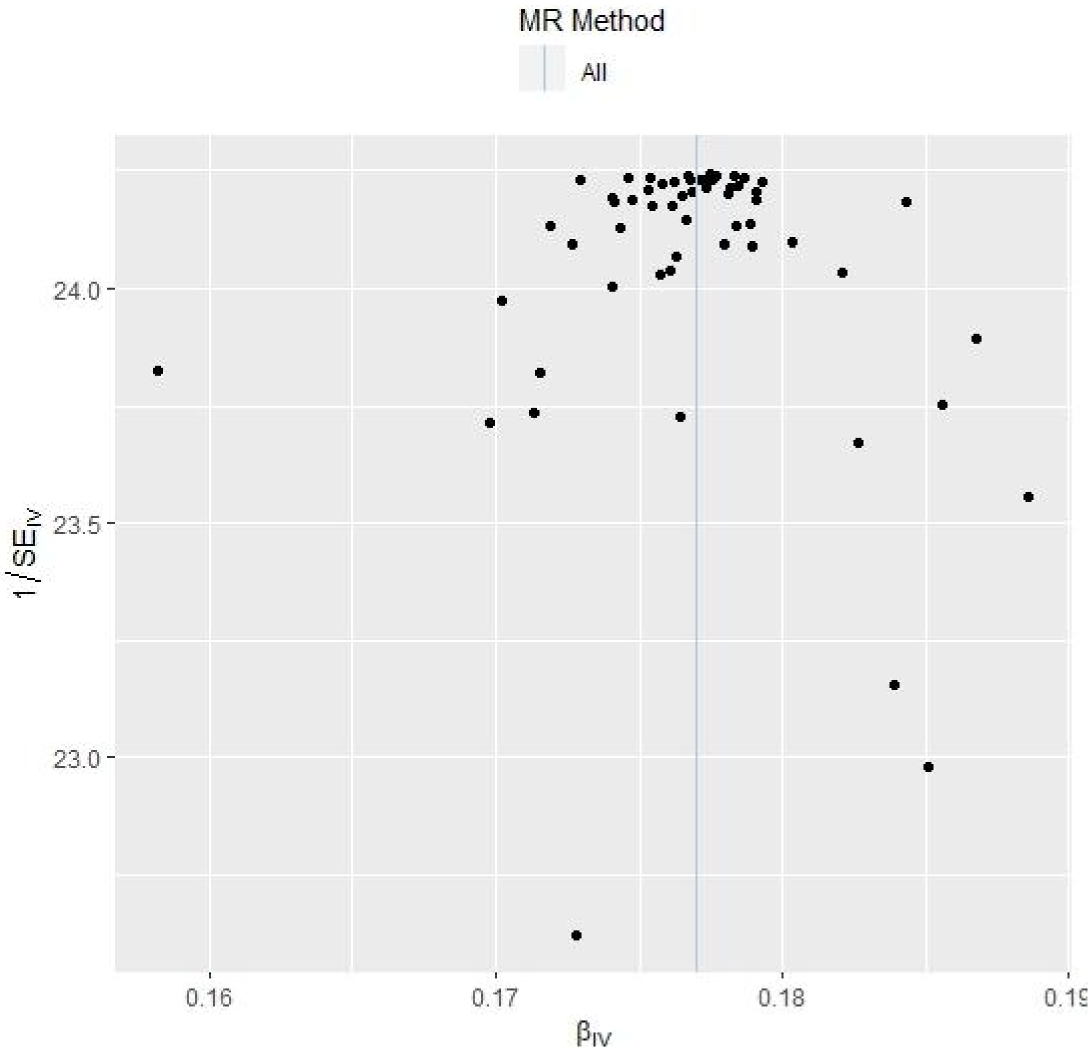

Supplemental Figure 15. Funnel plot of between LPL and Sleep apnea syndrome DMR. DMR, drug target Mendelian randomization.

PCSK9 & CHD scatter plot

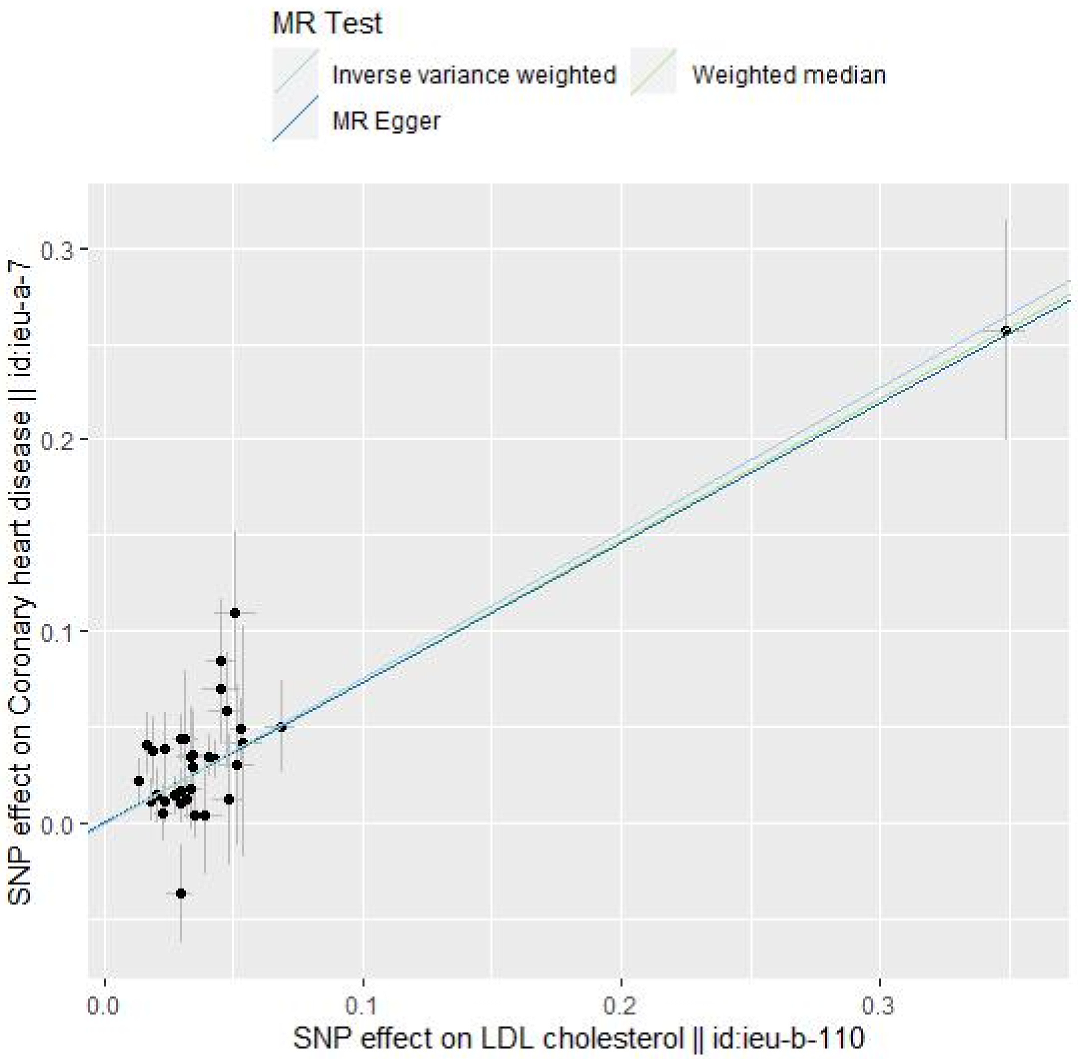

Supplemental Figure 16 Scatter plot of between PCSK9 and Coronary Heart Disease DMR. DMR, drug target Mendelian randomization.

PCSK9 & CHD leave-one-out analysis

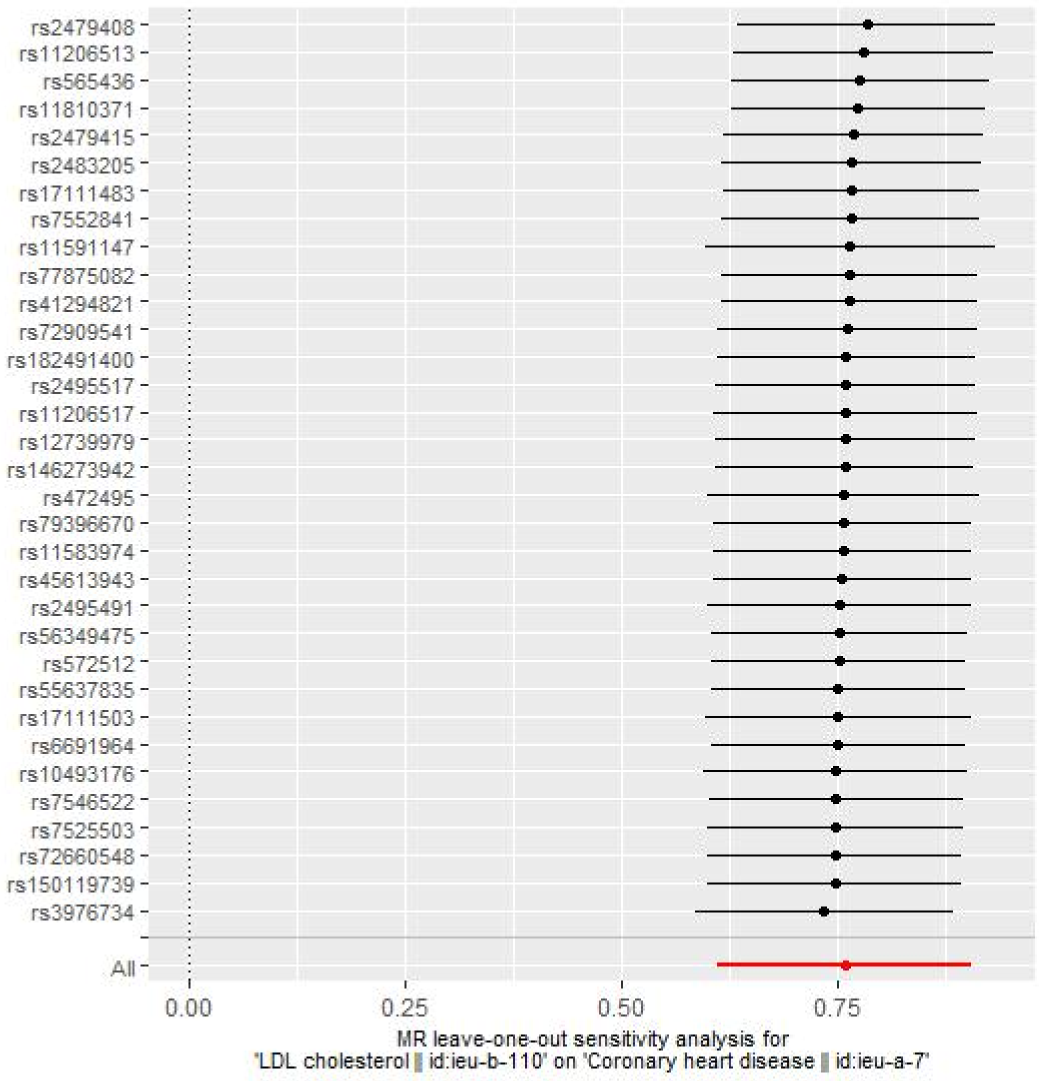

Supplemental Figure 17. PCSK9 and Coronary Heart Disease DMR leave-one-out analysis. DMR, drug target Mendelian randomization.

PCSK9 & CHD funnel plot

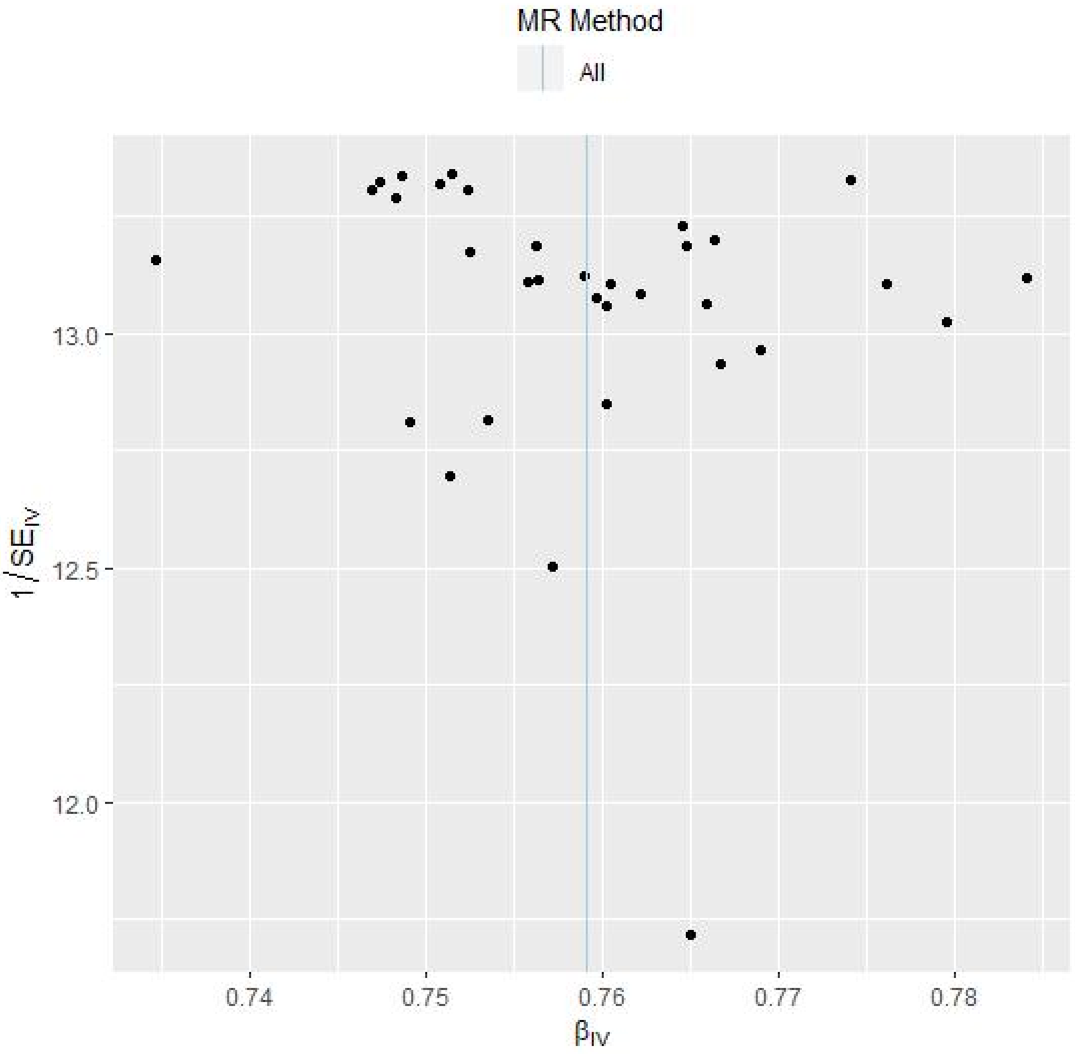

Supplemental Figure 18. Funnel plot of between PCSK9 and Coronary Heart Disease DMR. DMR, drug target Mendelian randomization

HMGCR & CHD scatter plot

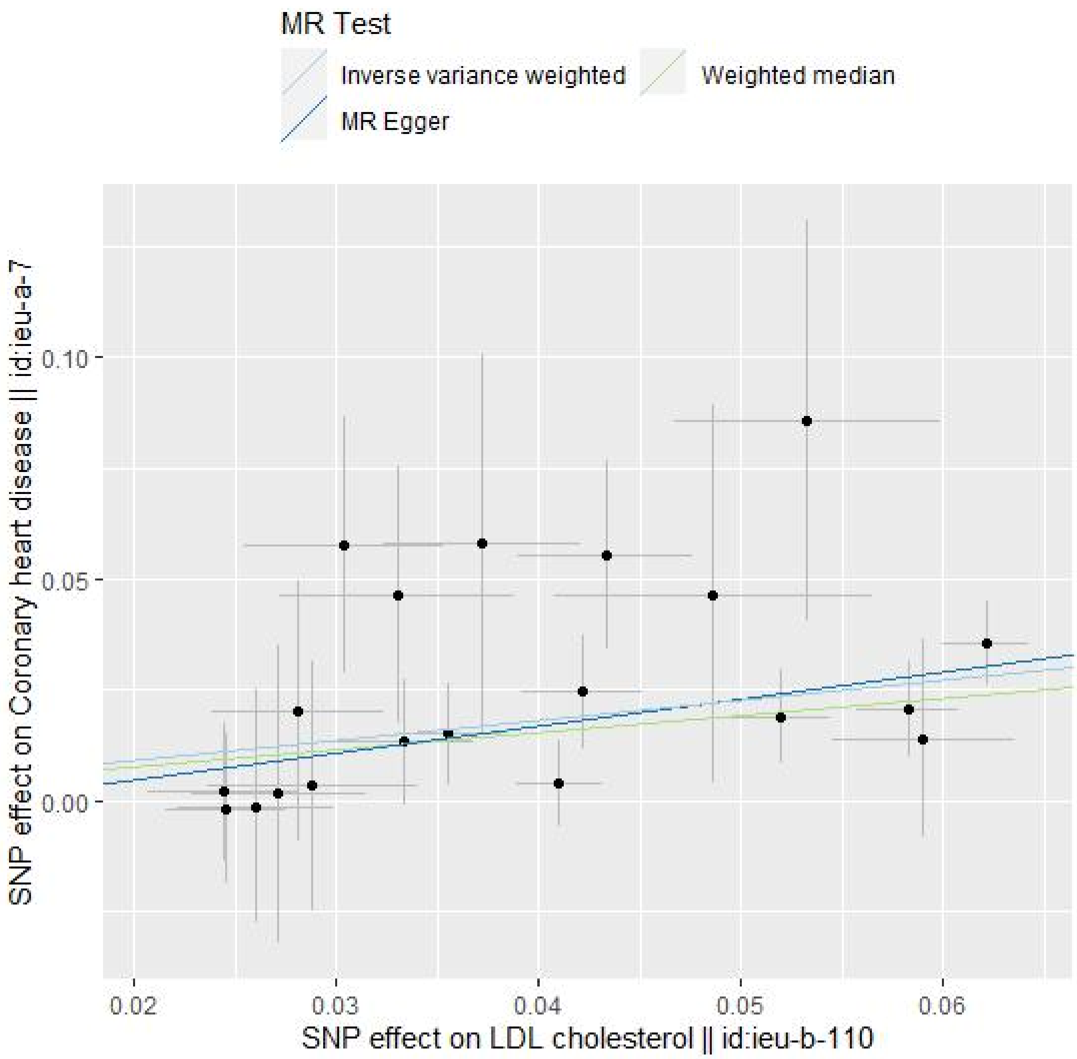

Supplemental Figure 19. Scatter plot of between HMGCR and Coronary Heart Disease DMR. DMR, drug target Mendelian randomization.

HMGCR & CHD leave-one-out analysis

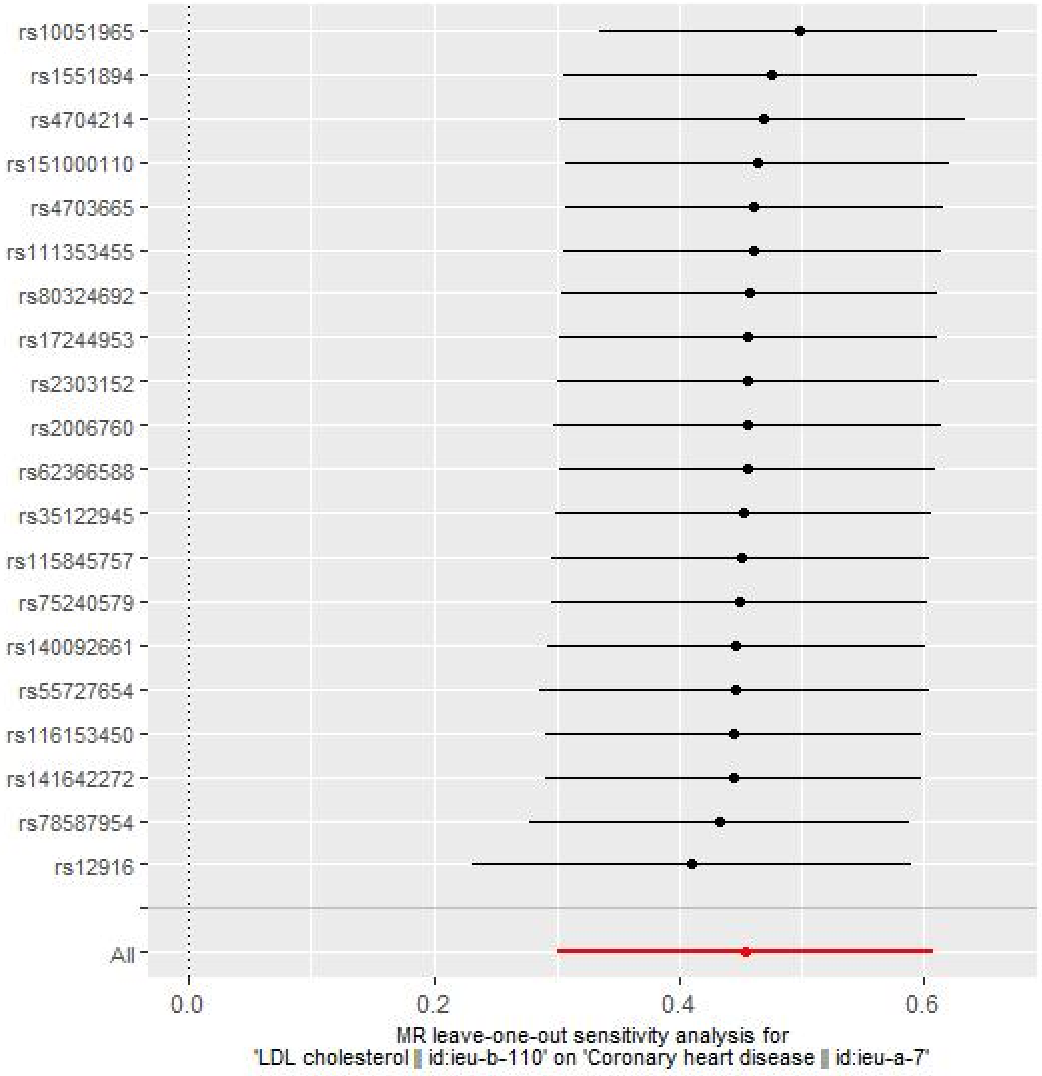

Supplemental Figure 20. HMGCR and Coronary Heart Disease DMR leave-one-out analysis. DMR, drug target Mendelian randomization.

HMGCR & CHD funnel plot

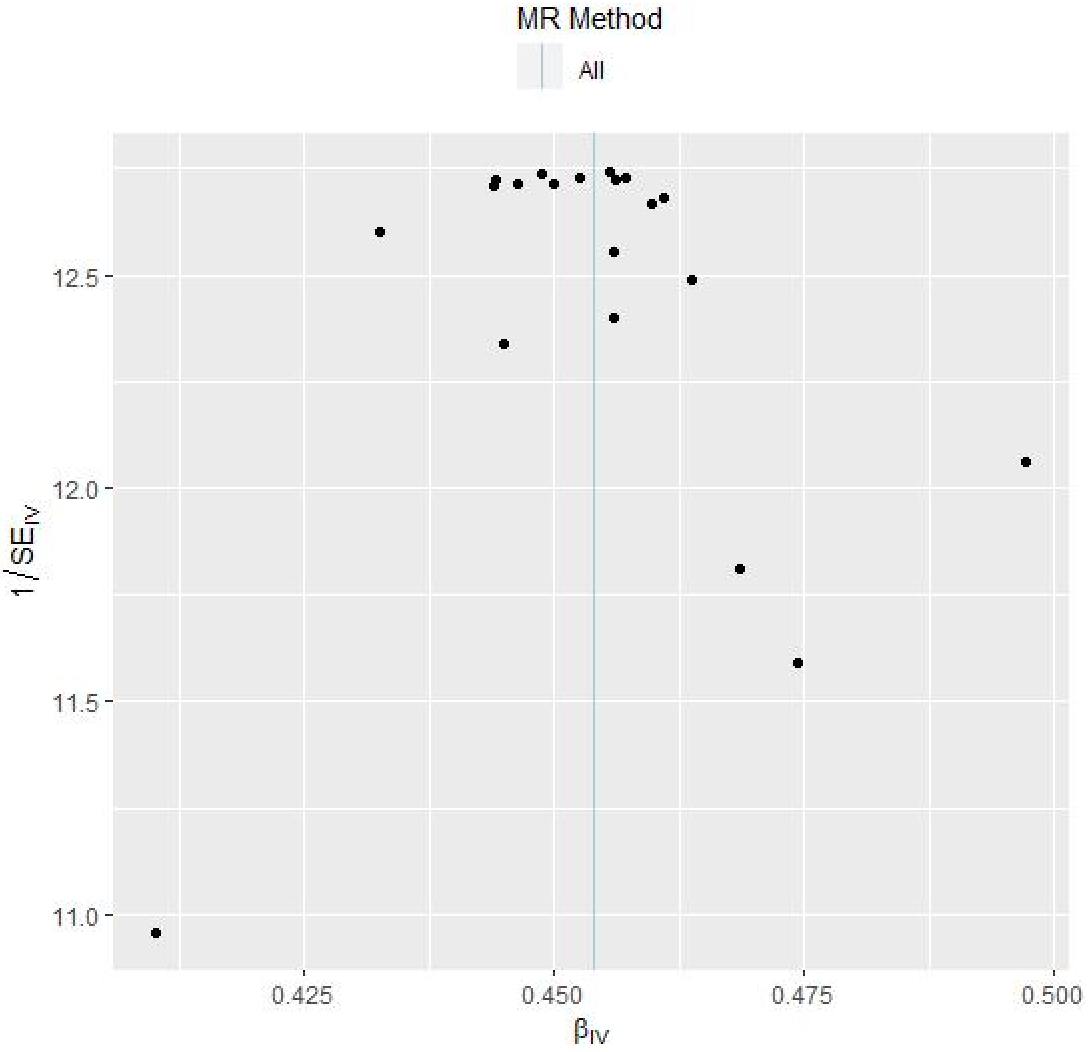

Supplemental Figure 21. Funnel plot of between HMGCR and Coronary Heart Disease DMR. DMR, drug target Mendelian randomization.

NPC1L1 & CHD funnel plot

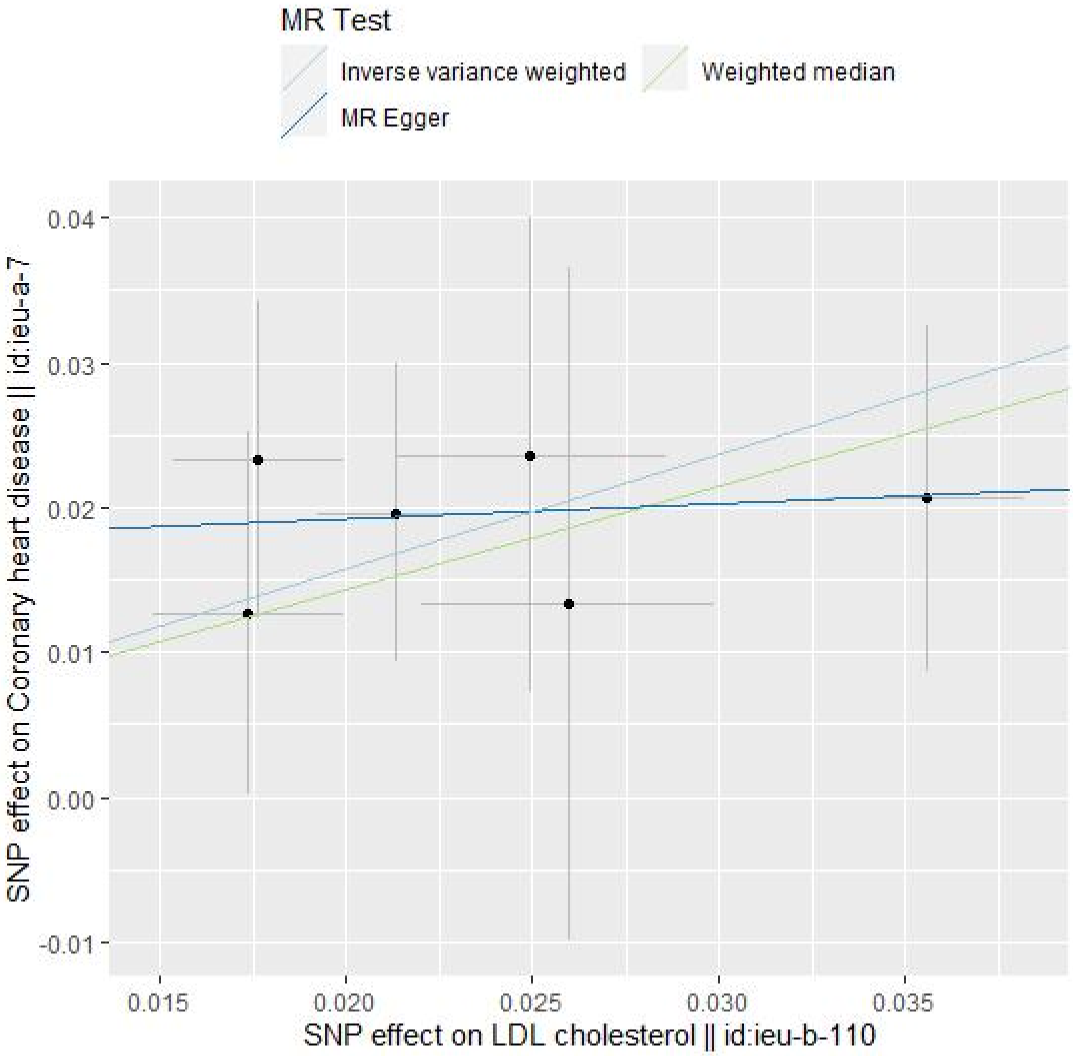

Supplemental Figure 22. Scatter plot of between NPC1L1 and Coronary Heart Disease DMR. DMR, drug target Mendelian randomization.

NPC1L1 & CHD leave-one-out analysis

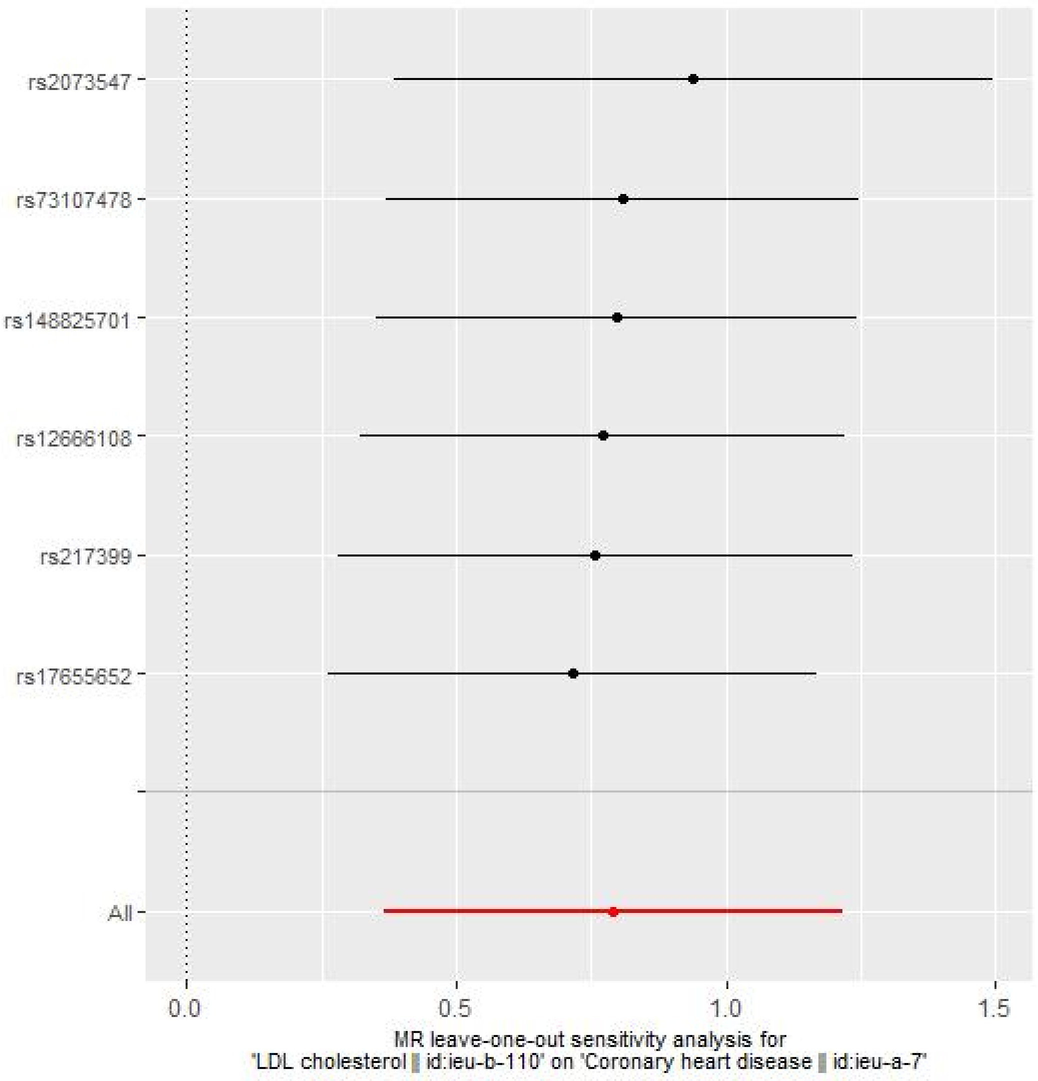

Supplemental Figure 23. NPC1L1 and Coronary Heart Disease DMR leave-one-out analysis. DMR, drug target Mendelian randomization.

NPC1L1 & CHD funnel plot

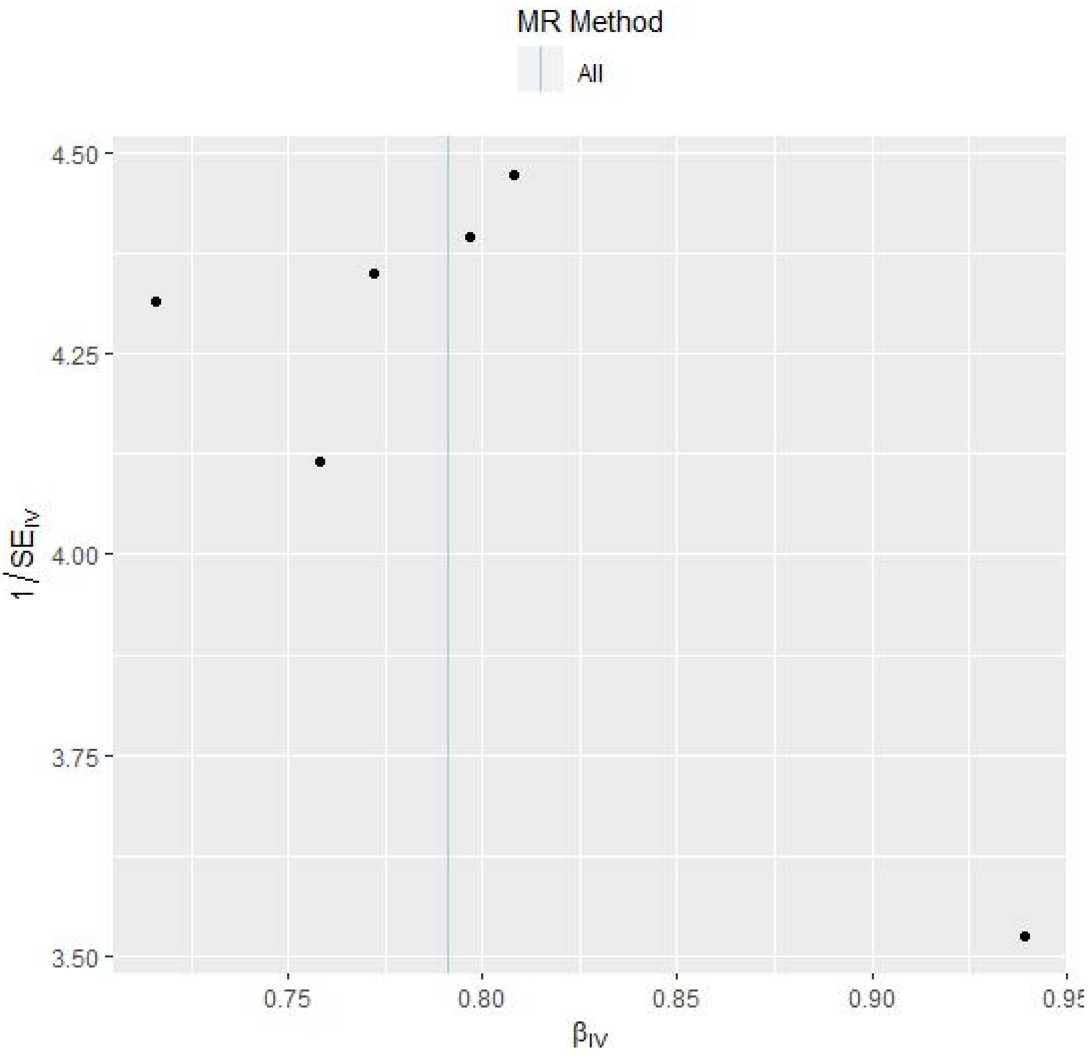

Supplemental Figure 24. Funnel plot of between NPC1L1 and Coronary Heart Disease DMR. DMR, drug target Mendelian randomization.

LPL & CHD scatter plot

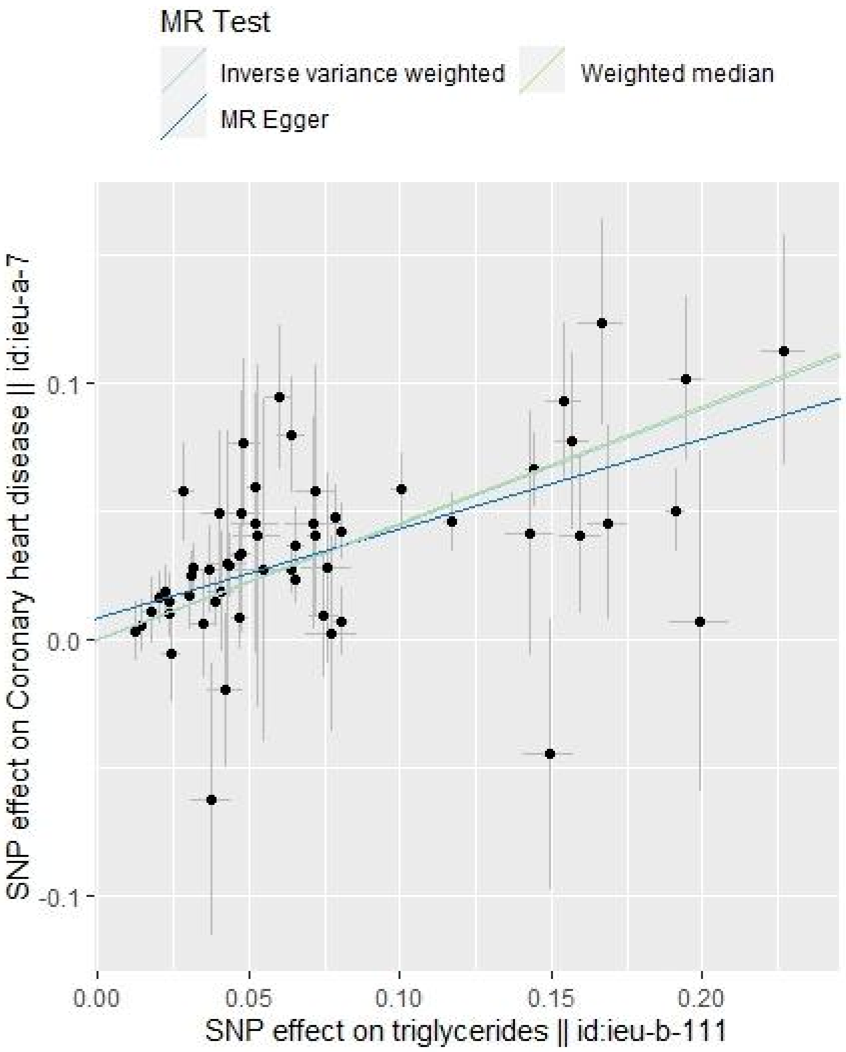

Supplemental Figure 25. Scatter plot of between LPL and Coronary Heart Disease DMR. DMR, drug target Mendelian randomization.

LPL & CHD leave-one-out analysis

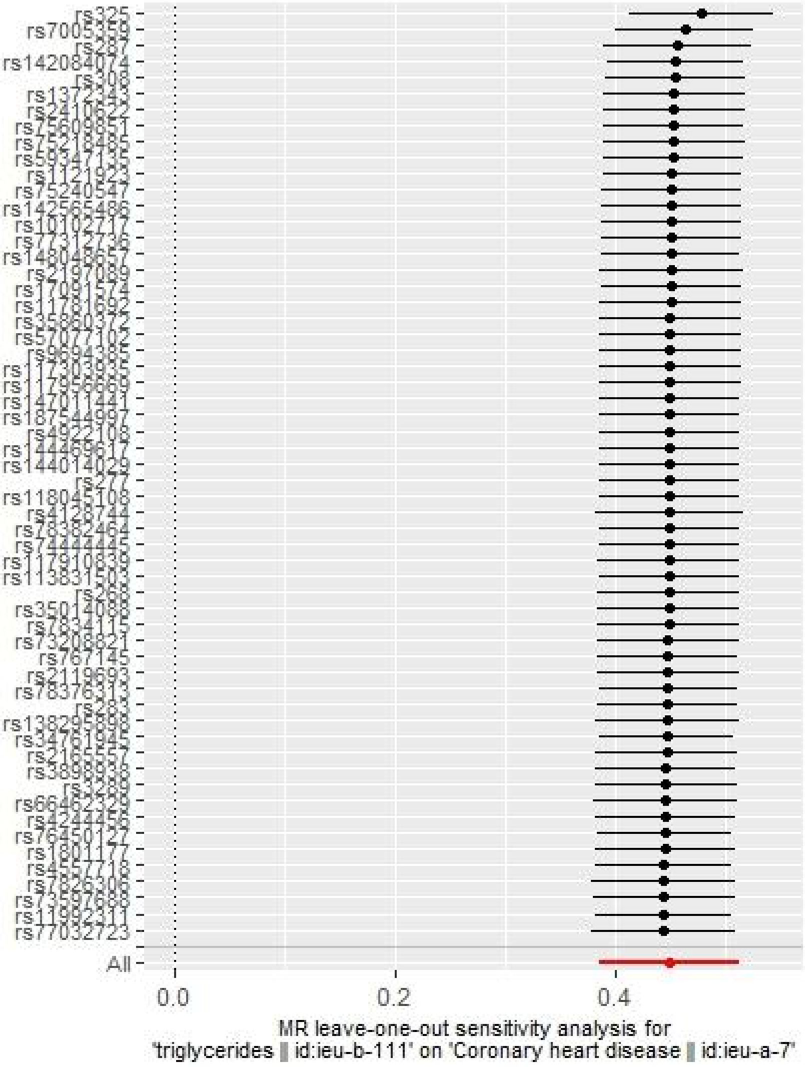

Supplemental Figure 26. LPL and Coronary Heart Disease DMR leave-one-out analysis. DMR, drug target Mendelian randomization

LPL & CHD funnel plot

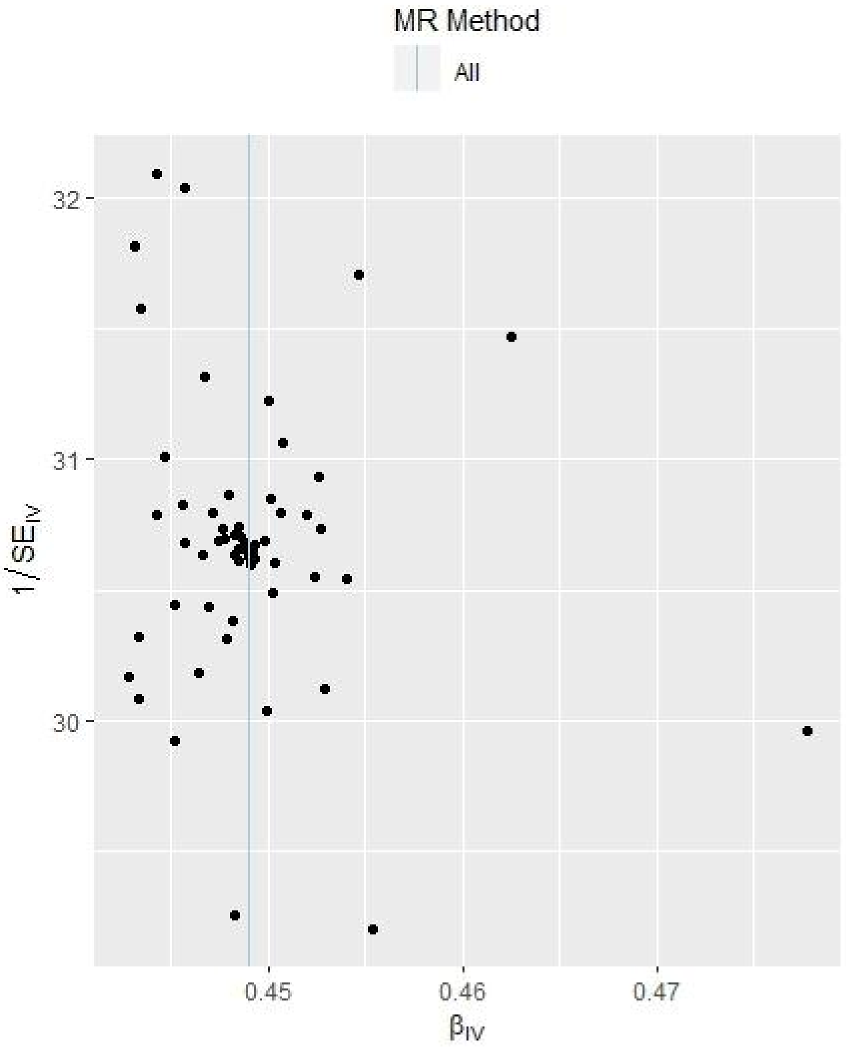

Supplemental Figure 27. Funnel plot of between LPL and Coronary Heart Disease DMR. DMR, drug target Mendelian randomization.

APOC3 & CHD scatter plot

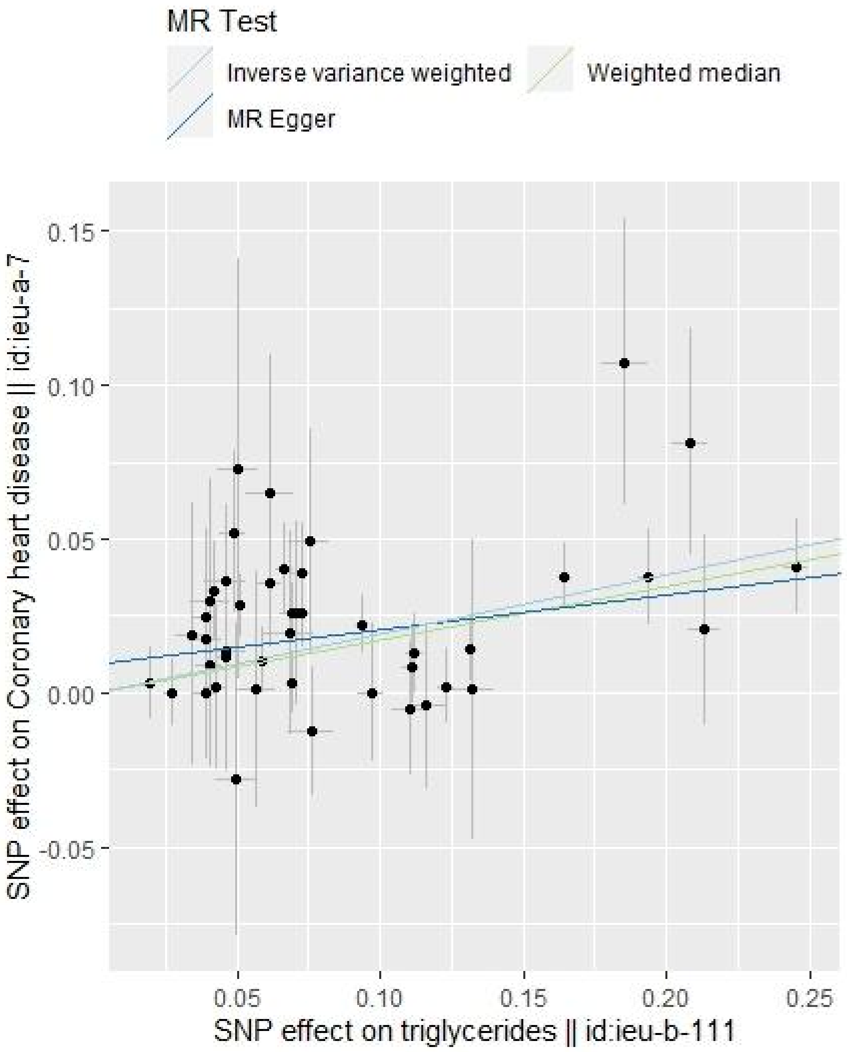

Supplemental Figure 28. Scatter plot of between APOC3 and Coronary Heart Disease DMR. DMR, drug target Mendelian randomization.

APOC3 & CHD leave-one-out analysis

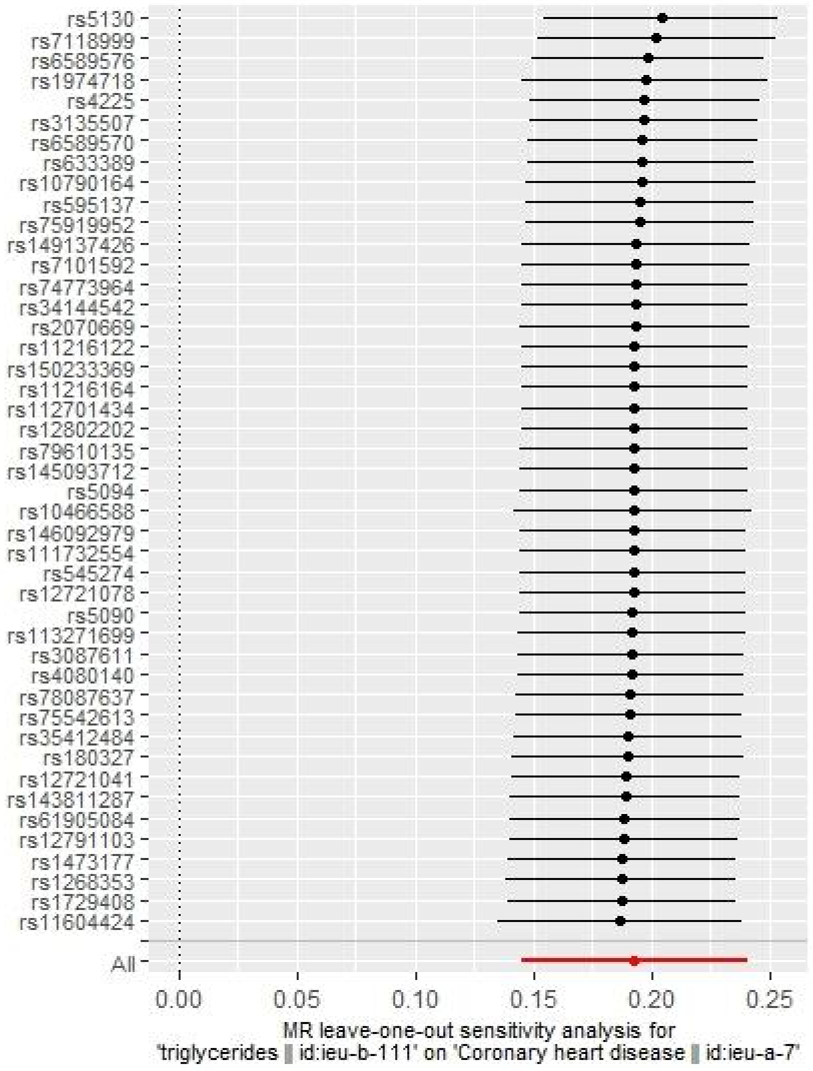

Supplemental Figure 29. APOC3 and Coronary Heart Disease DMR leave-one-out analysis. DMR, drug target Mendelian randomization.

APOC3 & CHD funnel plot

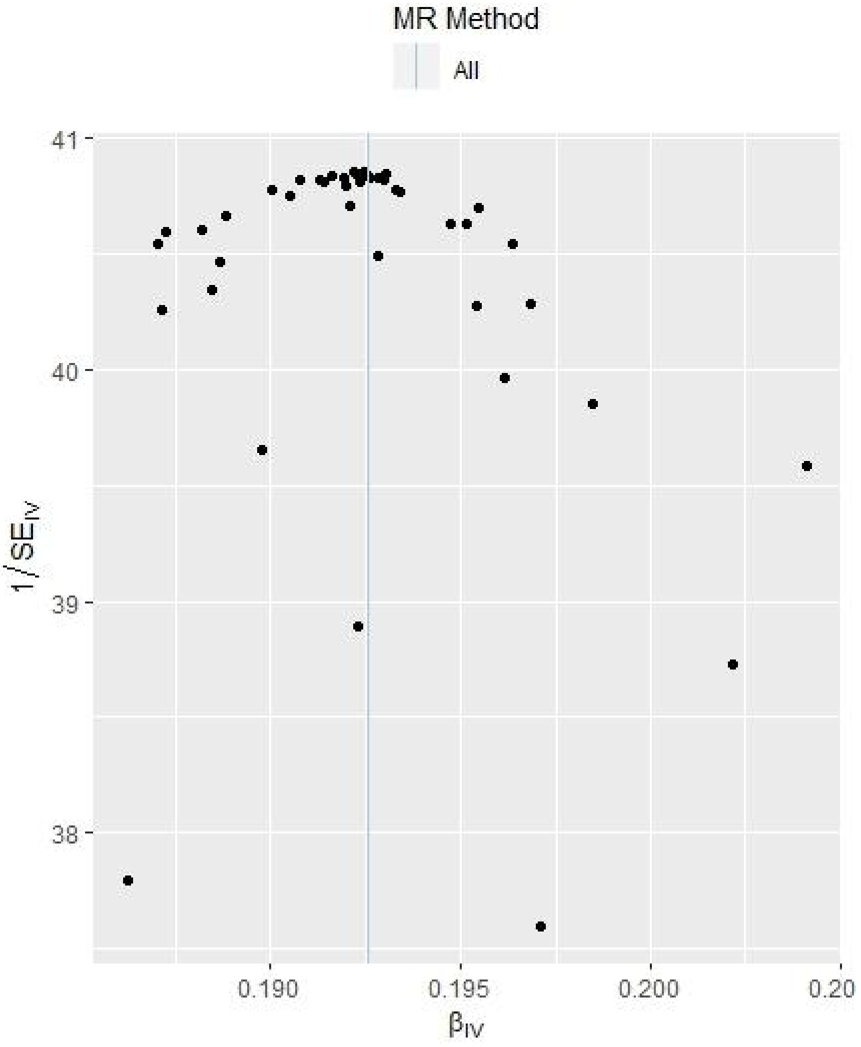

Supplemental Figure 30. Funnel plot of between APOC3 and Coronary Heart Disease DMR. DMR, drug target Mendelian randomization.

ANGPTL3 & CHD scatter plot

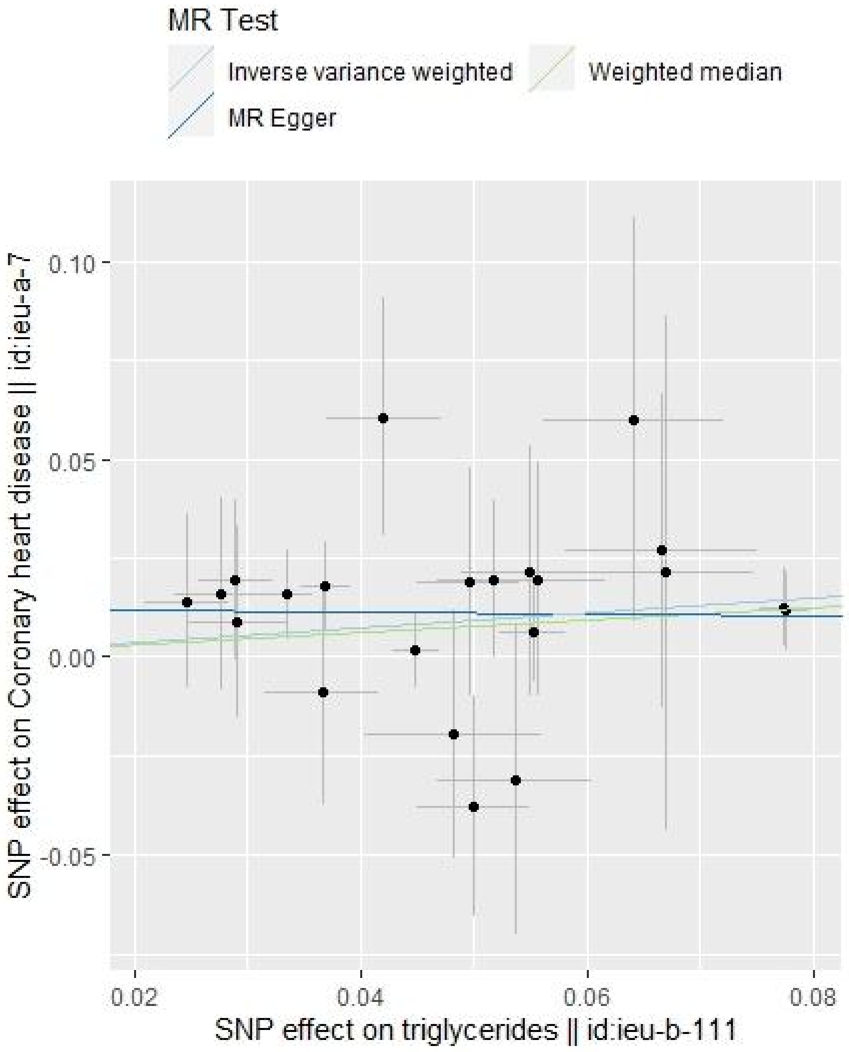

Supplemental Figure 31. Scatter plot of between ANGPTL3 and Coronary Heart Disease DMR. DMR, drug target Mendelian randomization.

ANGPTL3 & CHD leave-one-out analysis

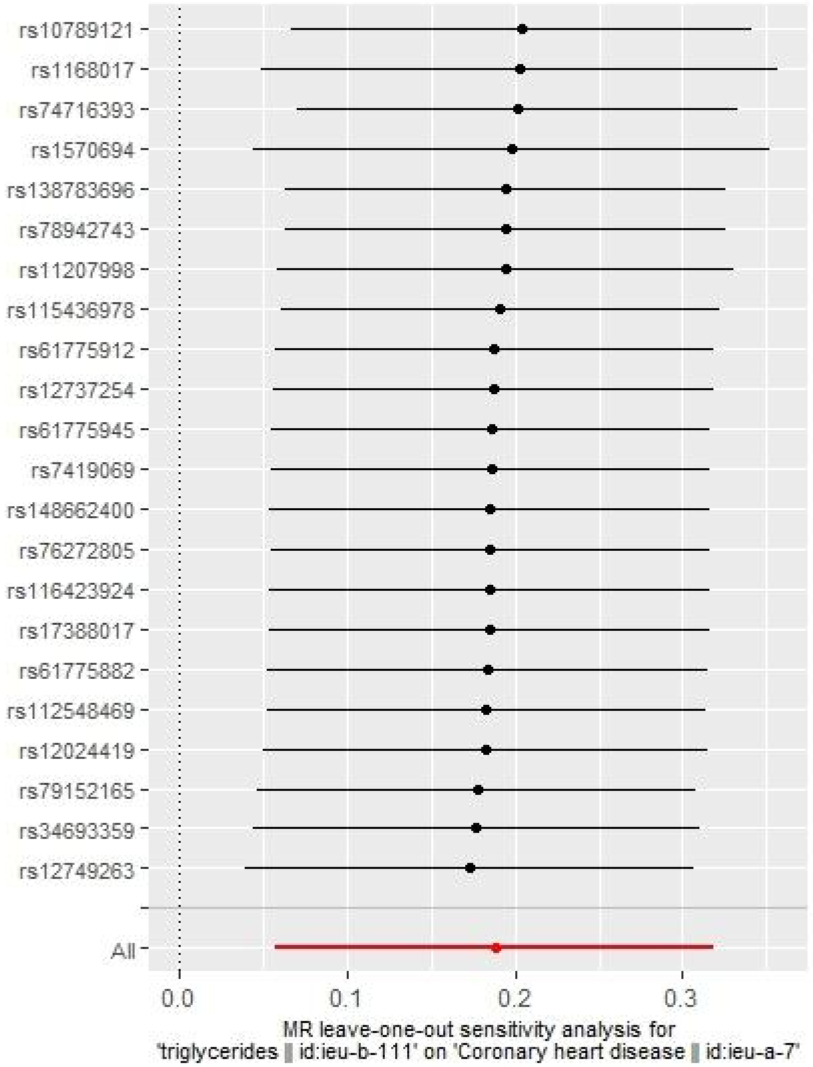

Supplemental Figure 32. ANGPTL3 and Coronary Heart Disease DMR leave-one-out analysis. DMR, drug target Mendelian randomization.

ANGPTL3 & CHD funnel plot

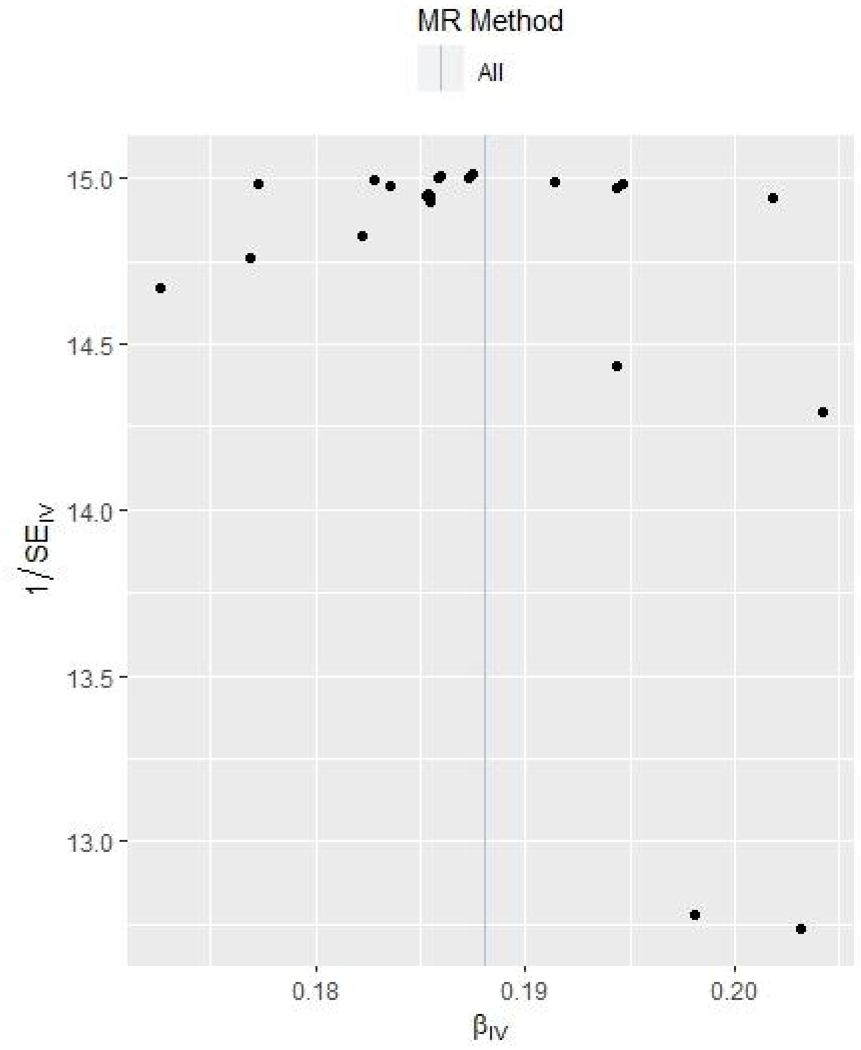

Supplemental Figure 33. Funnel plot of between ANGPTL3 and Coronary Heart Disease DMR. DMR, drug target Mendelian randomization.

CETP & CHD scatter plot

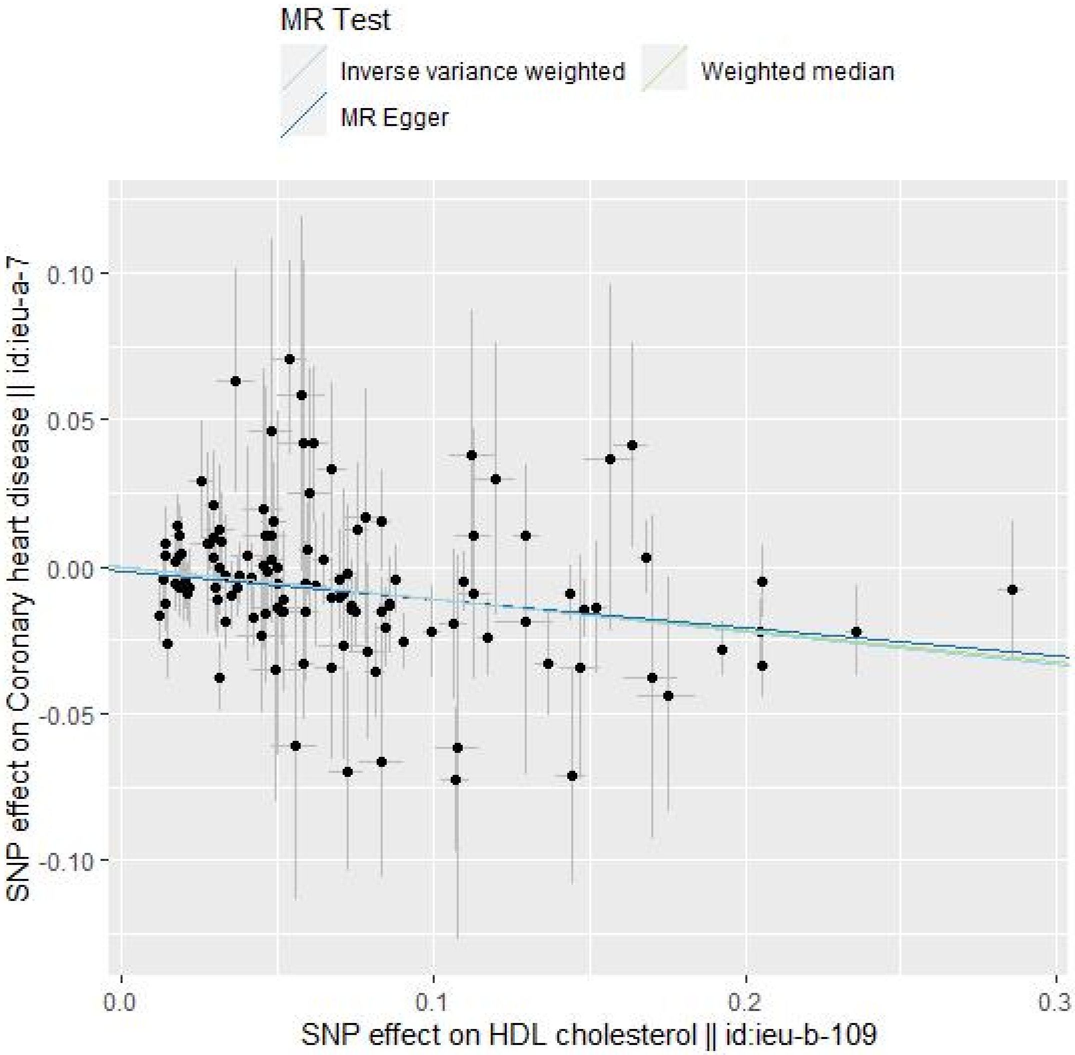

Supplemental Figure 34. Scatter plot of between CETP and Coronary Heart Disease DMR. DMR, drug target Mendelian randomization.

CETP & CHD leave-one-out analysis

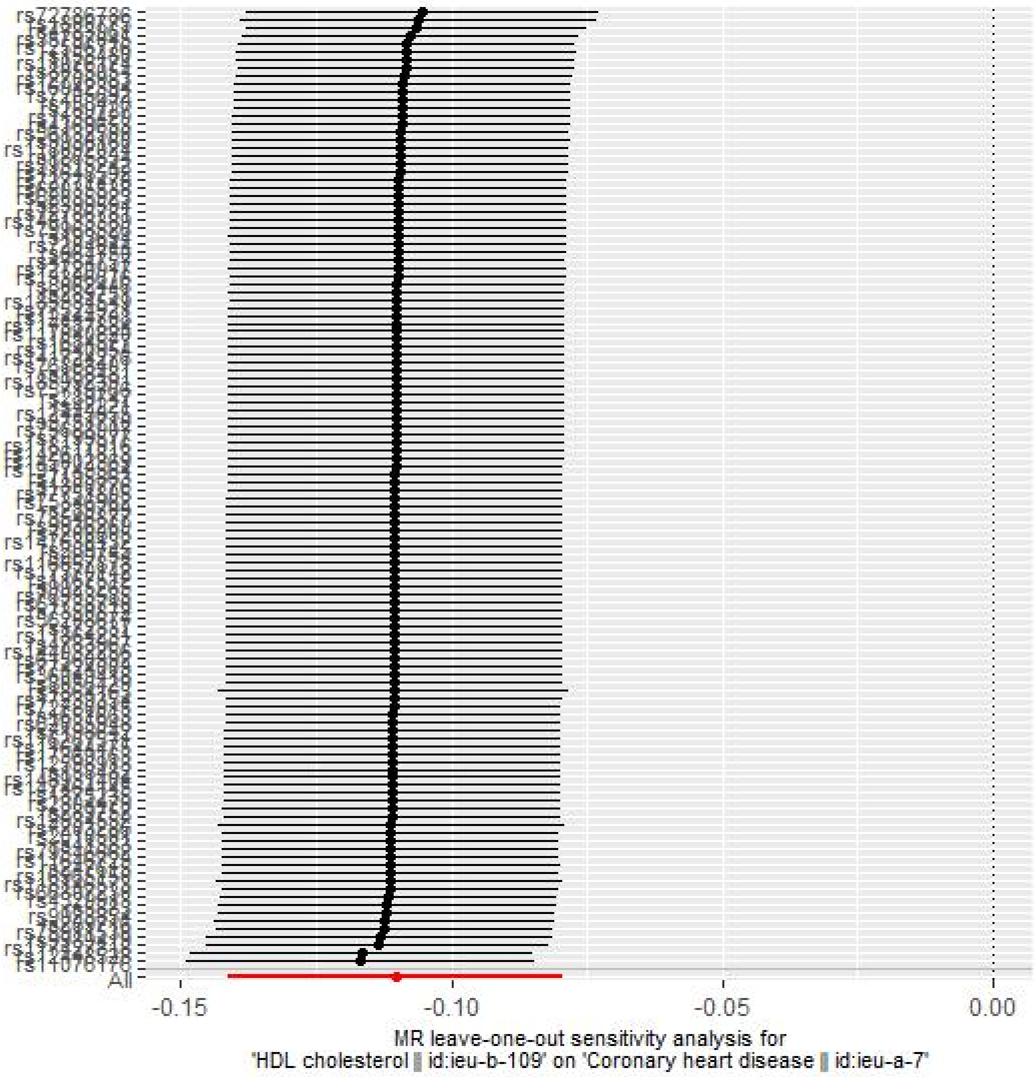

Supplemental Figure 35. CETP and Coronary Heart Disease DMR leave-one-out analysis. DMR, drug target Mendelian randomization.

CETP & CHD funnel plot

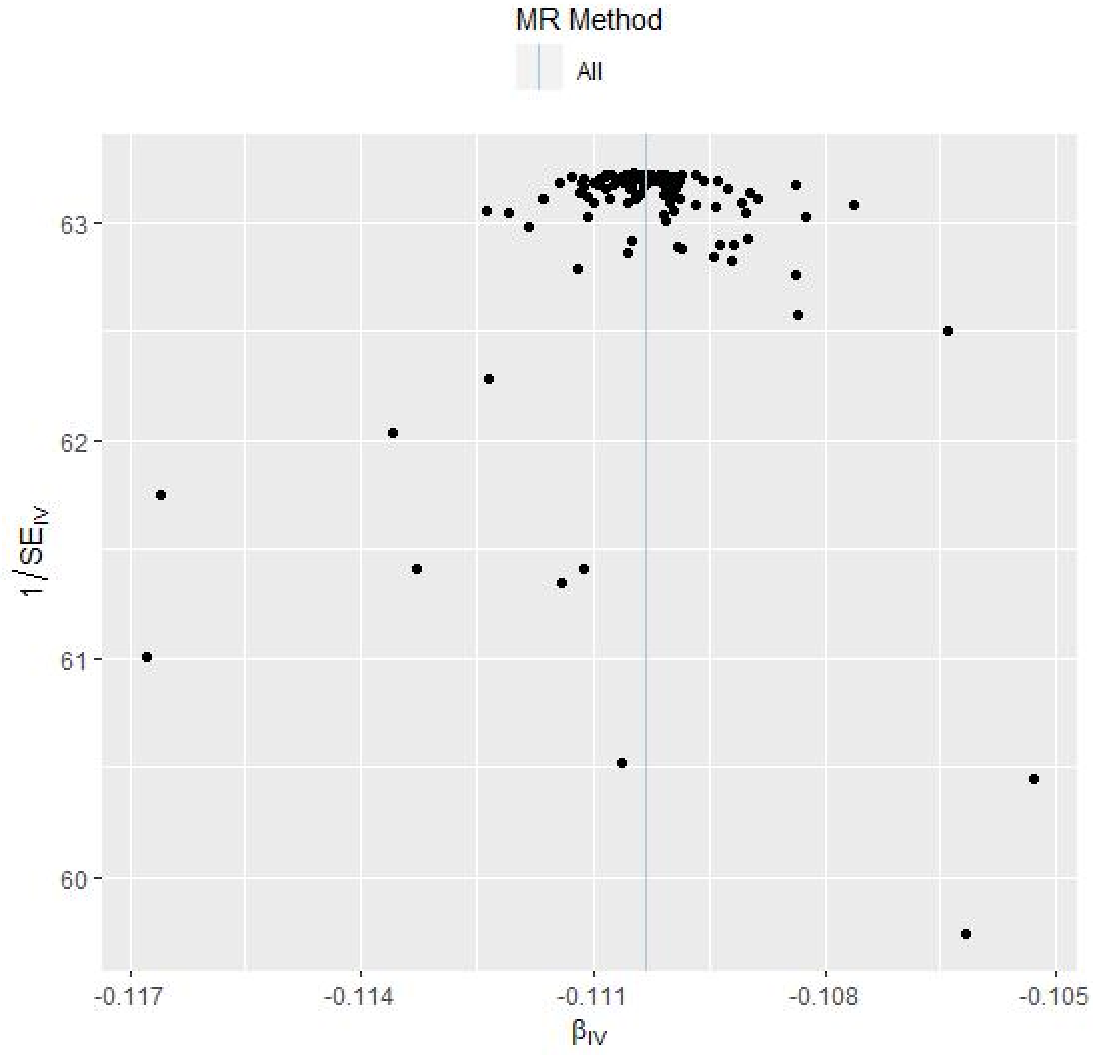

Supplemental Figure 36. Funnel plot of between CETP and Coronary Heart Disease DMR. DMR, drug target Mendelian randomization.

APOB & CHD scatter plot

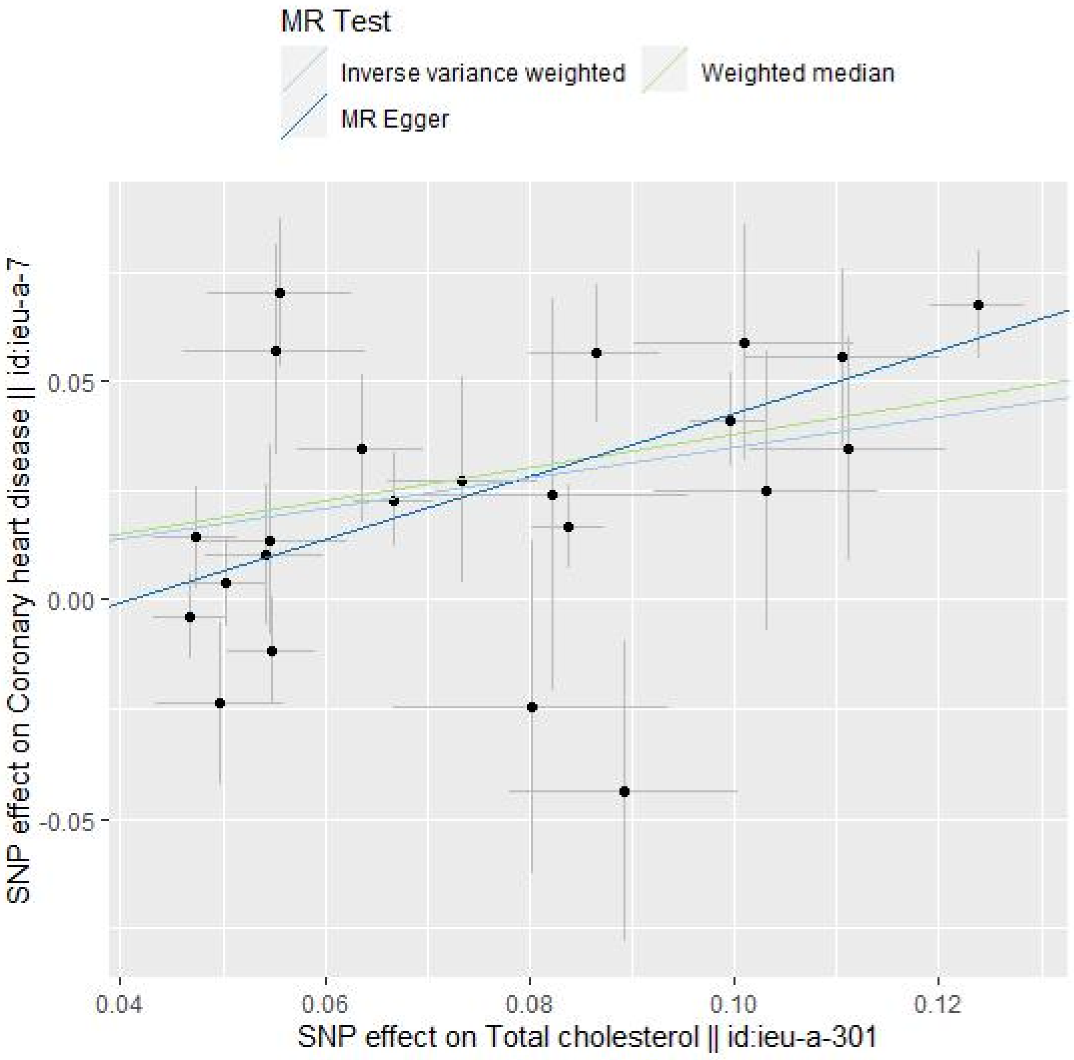

Supplemental Figure 37. Scatter plot of between APOB and Coronary Heart Disease DMR. DMR, drug target Mendelian randomization.

APOB & CHD leave-one-out analysis

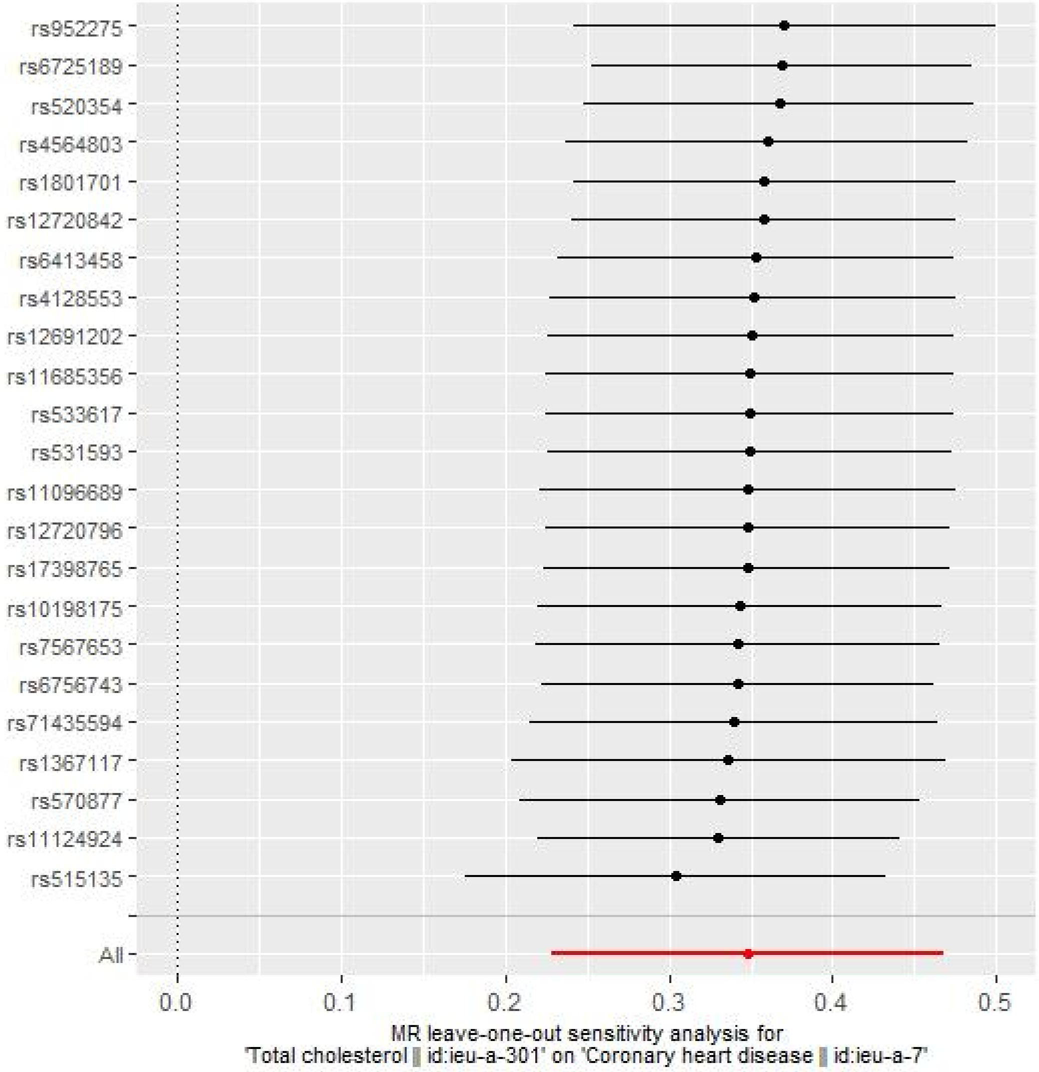

Supplemental Figure 38. CETP and Coronary Heart Disease DMR leave-one-out analysis. DMR, drug target Mendelian randomization.

APOB & CHD funnel plot

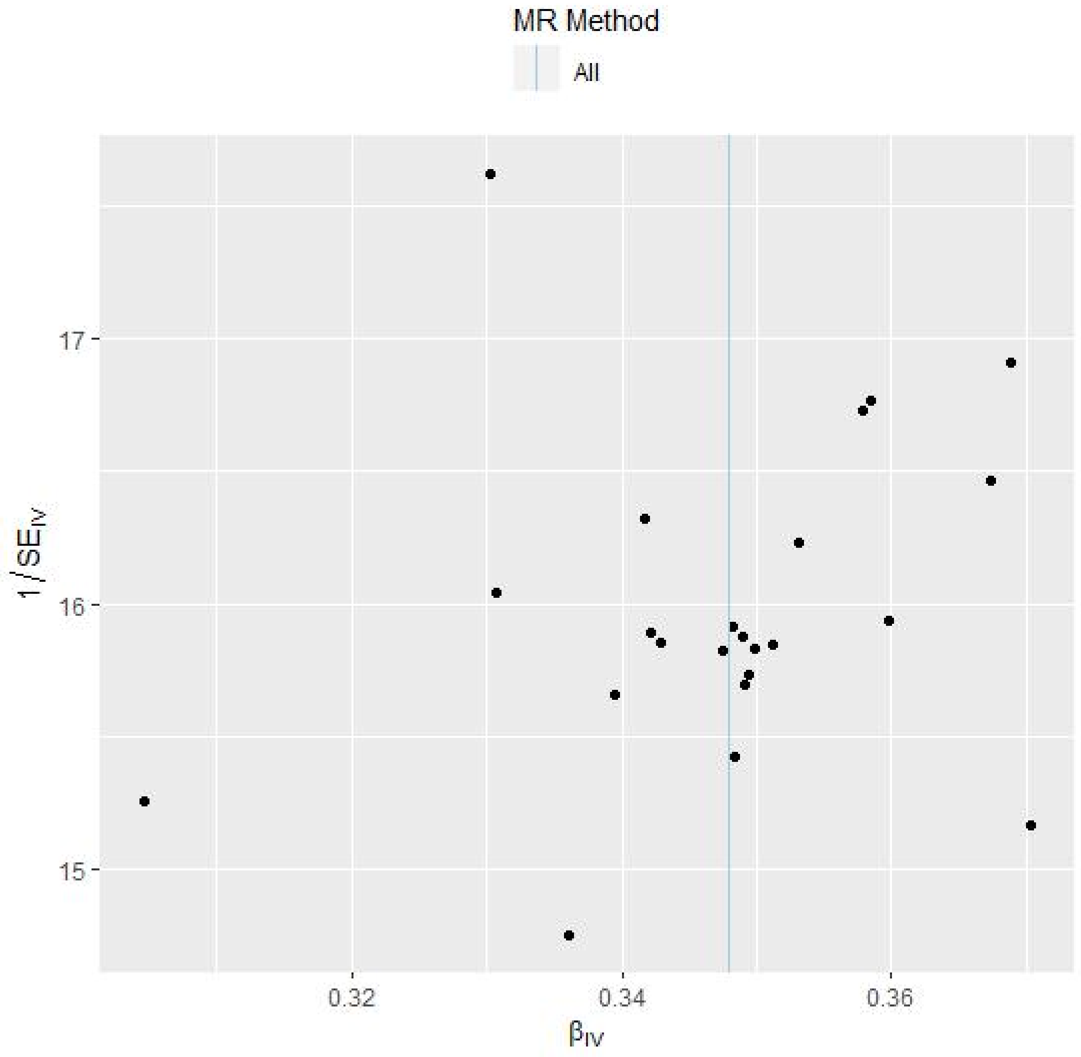

Supplemental Figure 39. Funnel plot of between APOB and Coronary Heart Disease DMR. DMR, drug target Mendelian randomization.

LDL-c & CHD scatter plot

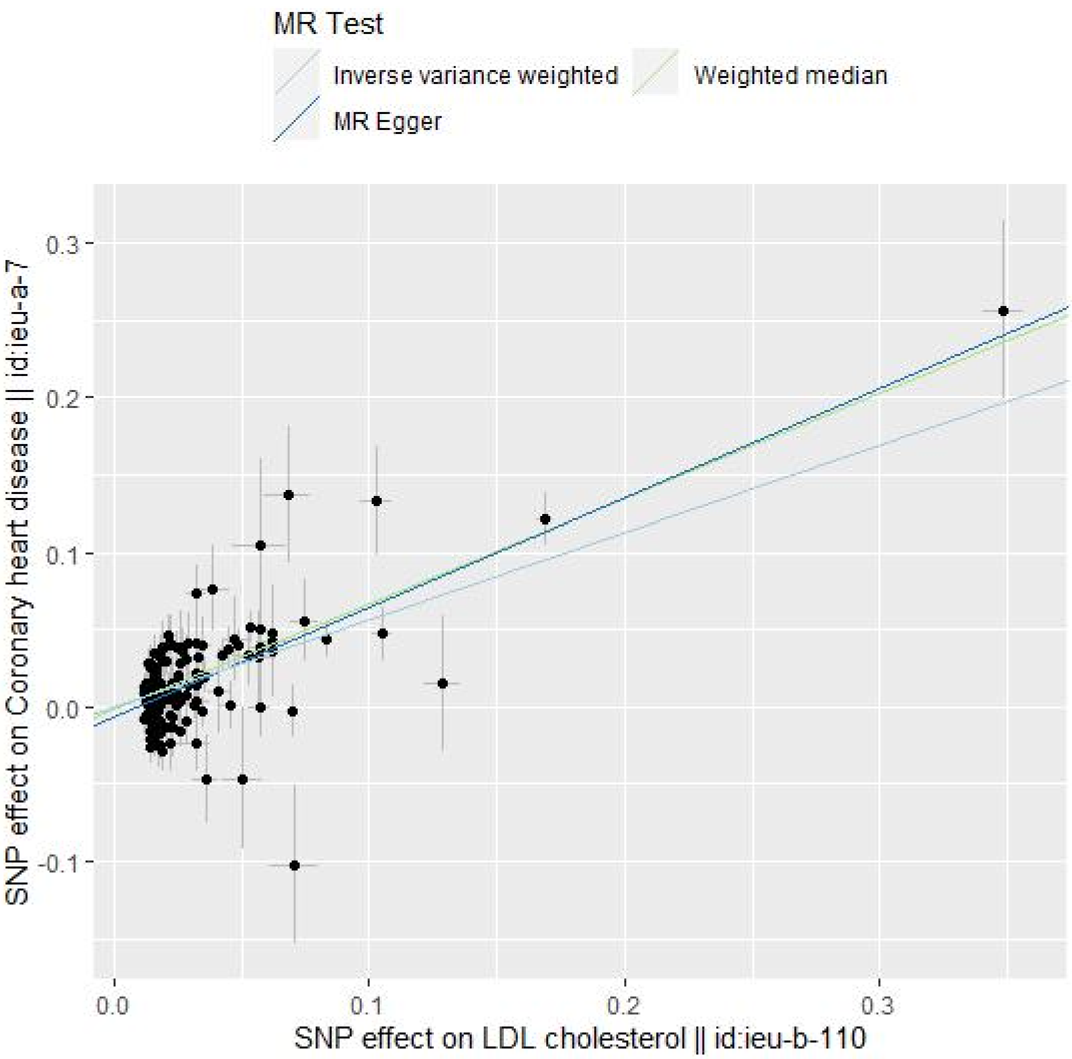

Supplemental Figure 40. Scatter plot of between serum LDL-c and Coronary Heart Disease TSMR analysis. LDL-c, low-density lipoprotein cholesterol; TSMR, two-sample Mendelian randomization.

LDL-c & CHD leave-one-out analysis

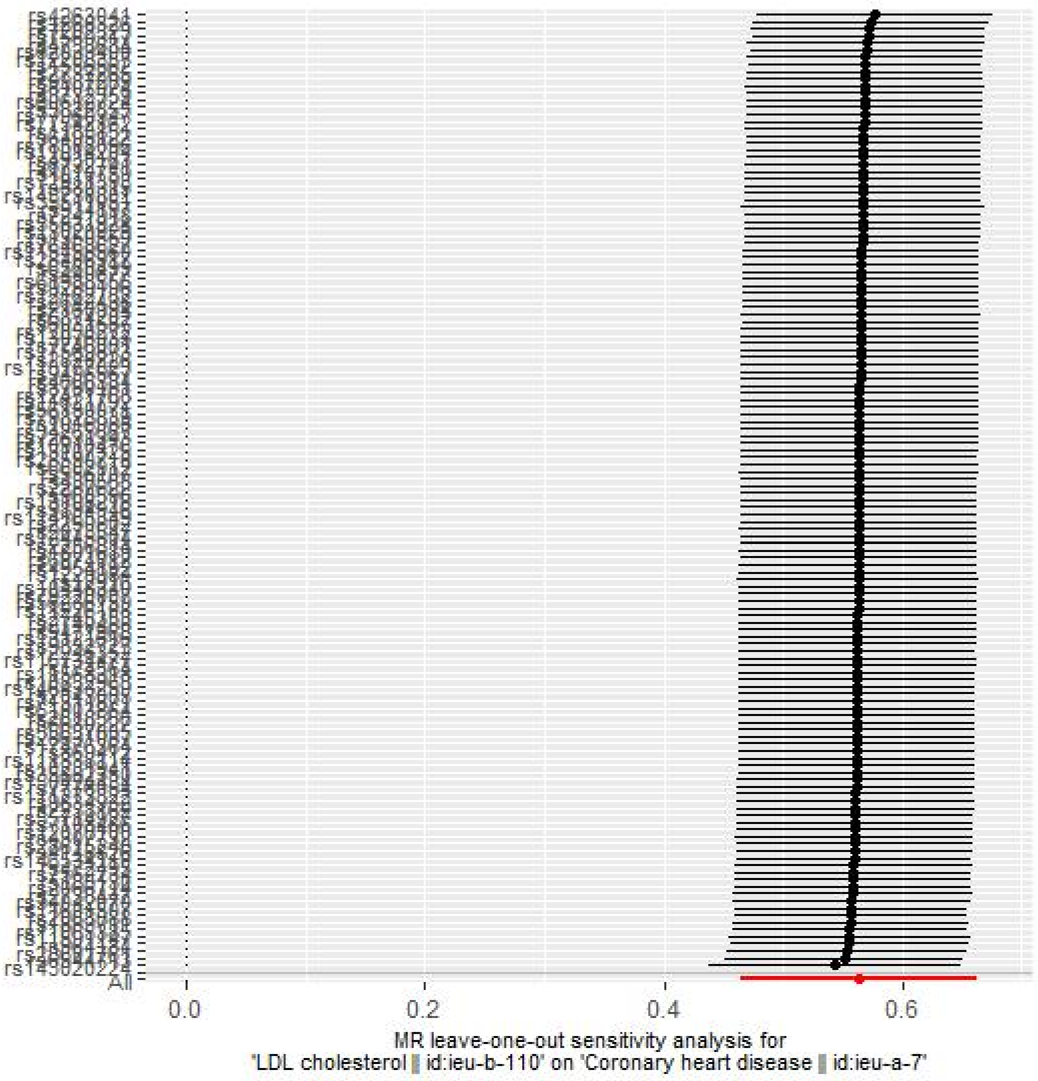

Supplemental Figure 41. Serum LDL-c and Coronary Heart Disease TSMR leave-one-out analysis. LDL-c, lowdensity lipoprotein cholesterol; TSMR, two-sample Mendelian randomization

LDL-c & CHD funnel plot

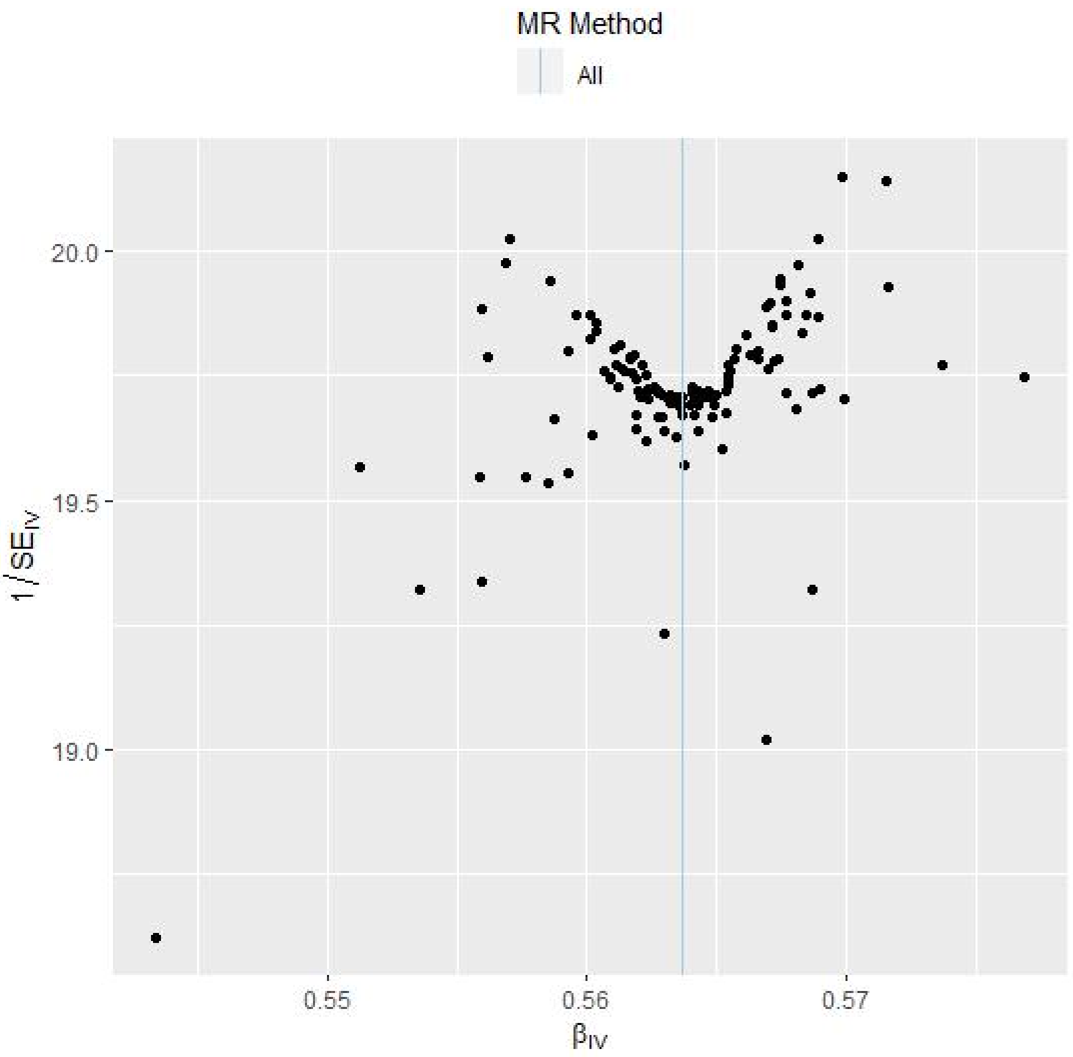

Supplemental Figure 42. Funnel plot of between serum LDL-c and Coronary Heart Disease TSMR analysis. LDL-c, low-density lipoprotein cholesterol; TSMR, two-sample Mendelian randomization.

HDL-c & CHD scatter plot

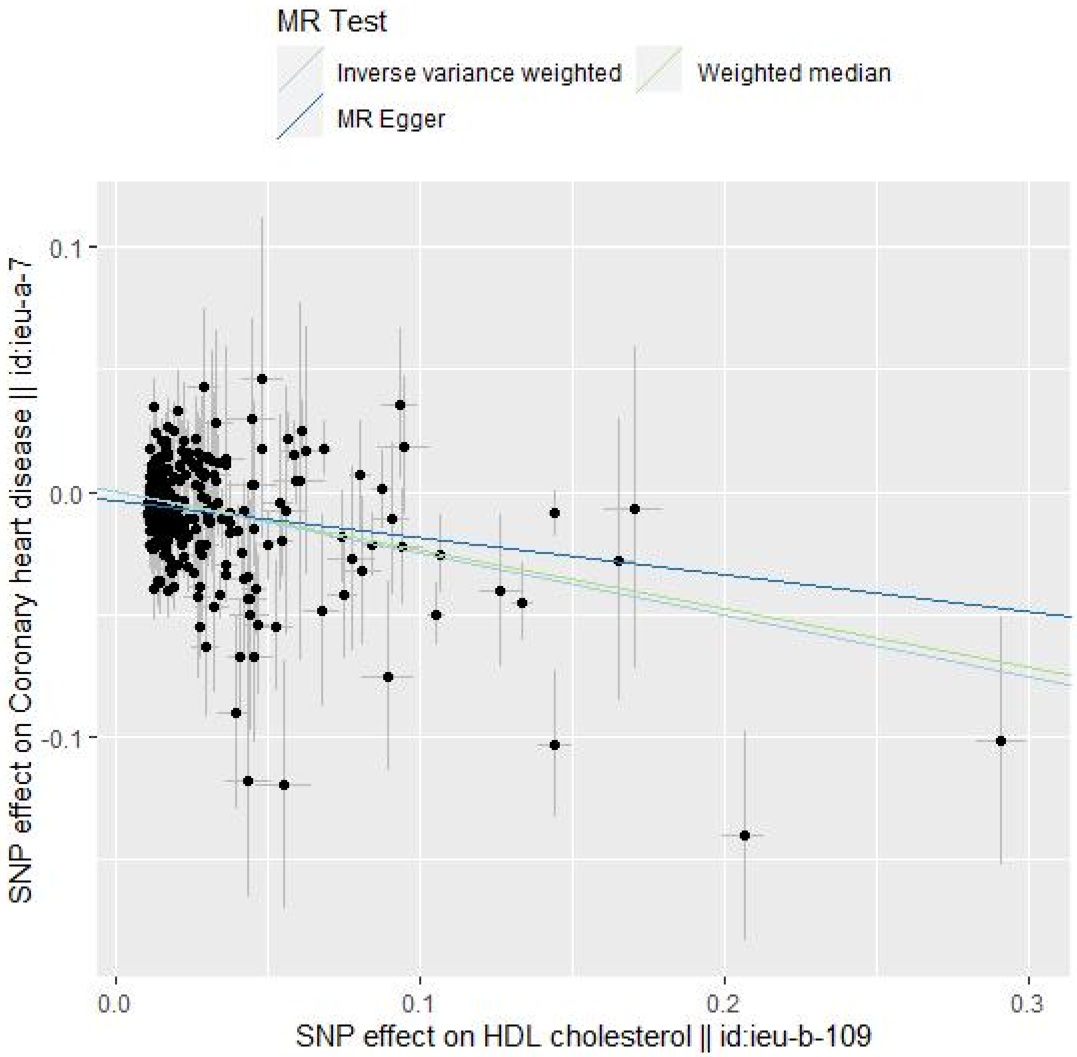

Supplemental Figure 43. Scatter plot of between serum HDL-c and Coronary Heart Disease TSMR analysis. LDL-c, low-density lipoprotein cholesterol; TSMR, two-sample Mendelian randomization.

HDL-c & CHD leave-one-out analysis

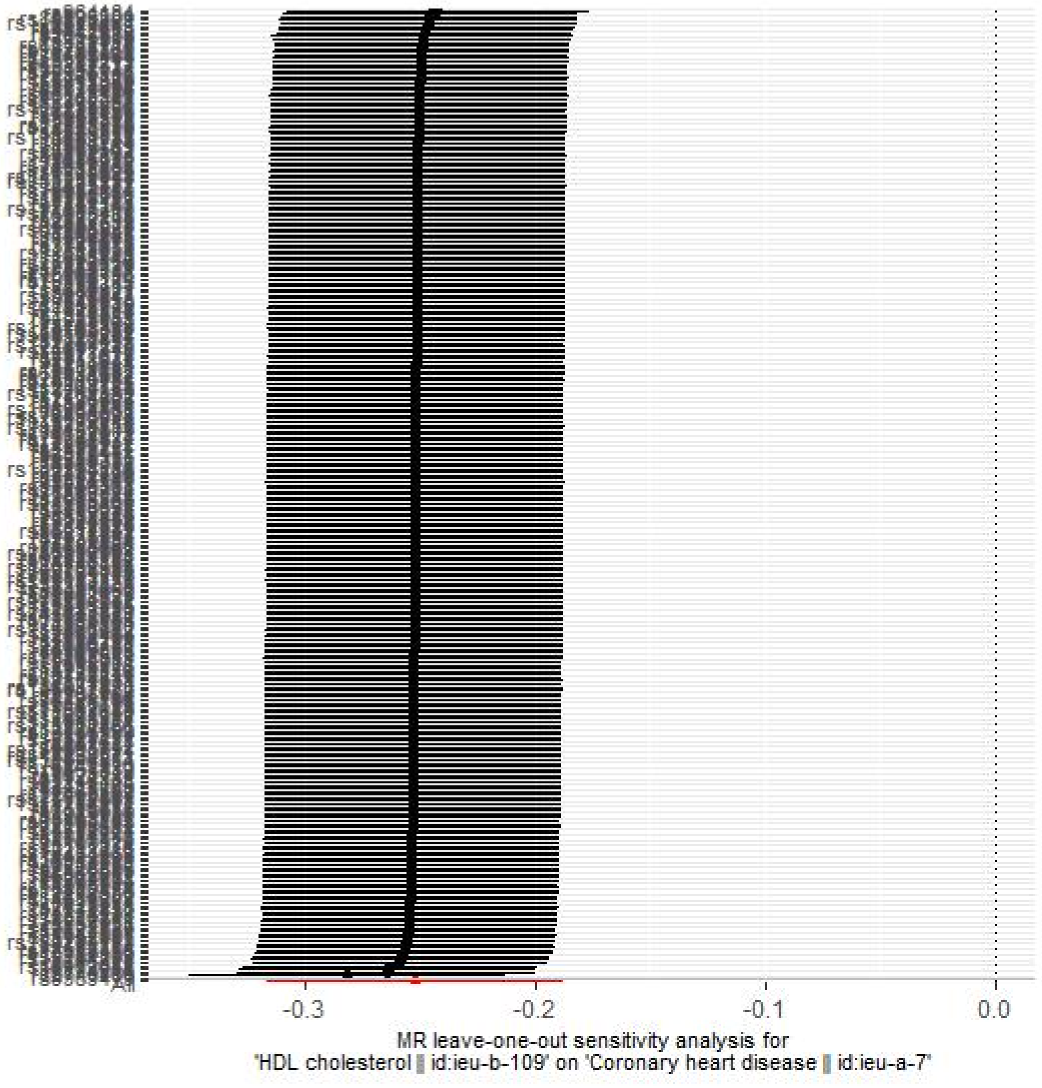

Supplemental Figure 44. Serum HDL-c and Coronary Heart Disease TSMR leave-one-out analysis. LDL-c, lowdensity lipoprotein cholesterol; TSMR, two-sample Mendelian randomization

HDL-c & CHD funnel plot

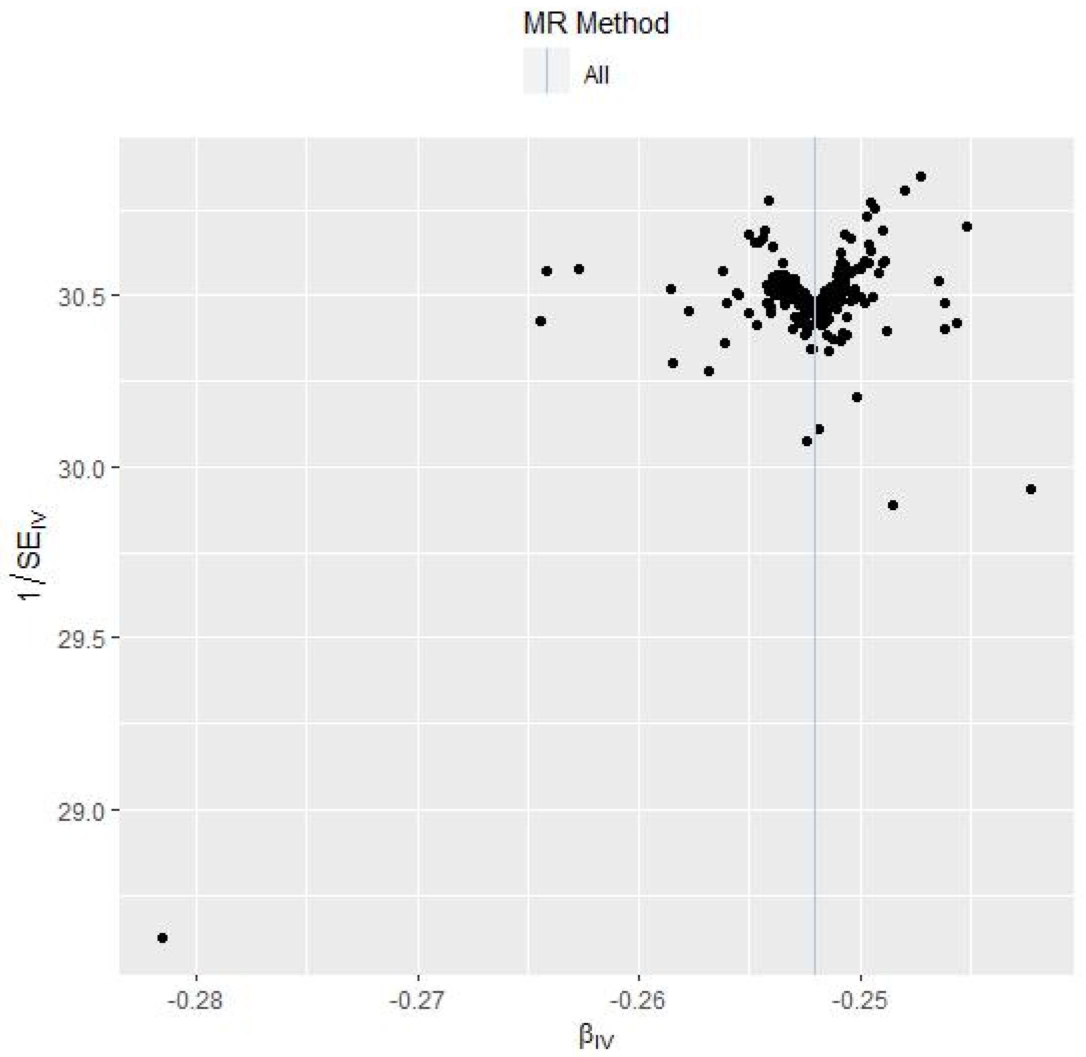

Supplemental Figure 45. Funnel plot of between serum HDL-c and Coronary Heart Disease TSMR analysis. LDL-c, low-density lipoprotein cholesterol; TSMR, two-sample Mendelian randomization.

TG & CHD scatter plot

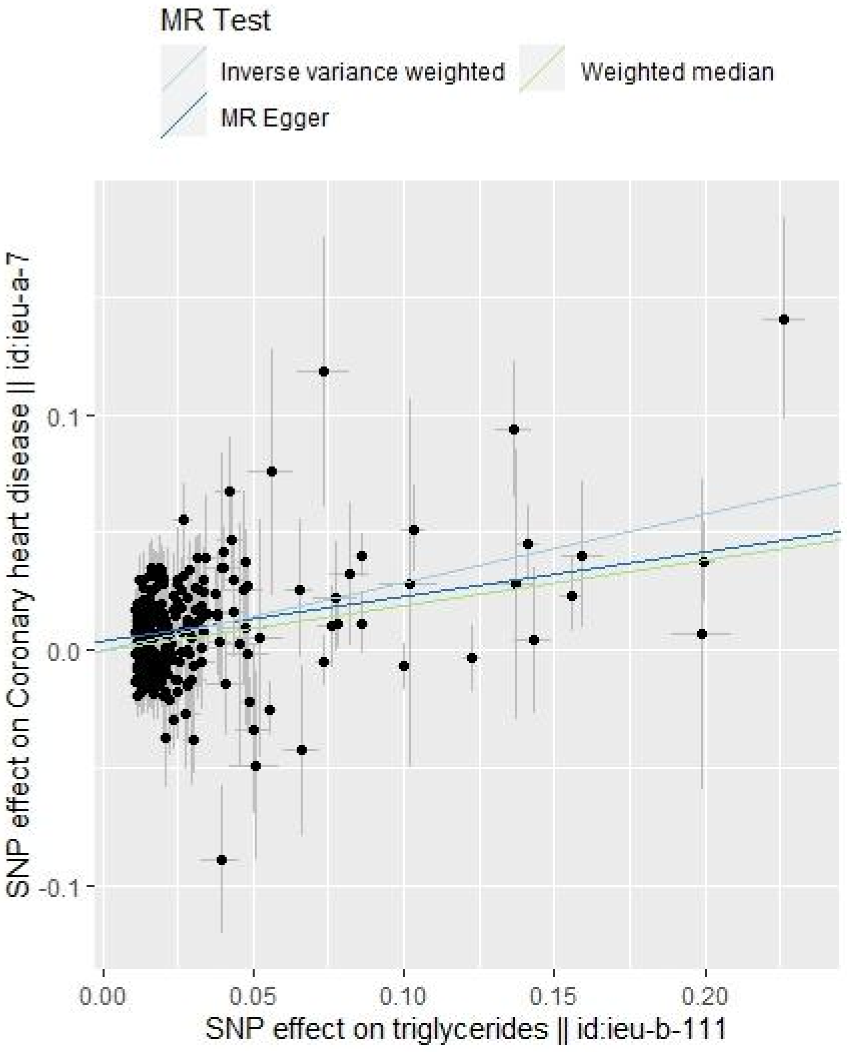

Supplemental Figure 46. Scatter plot of between serum TG and Coronary Heart Disease TSMR analysis. LDL-c, low-density lipoprotein cholesterol; TSMR, two-sample Mendelian randomization.

TG & CHD leave-one-out analysis

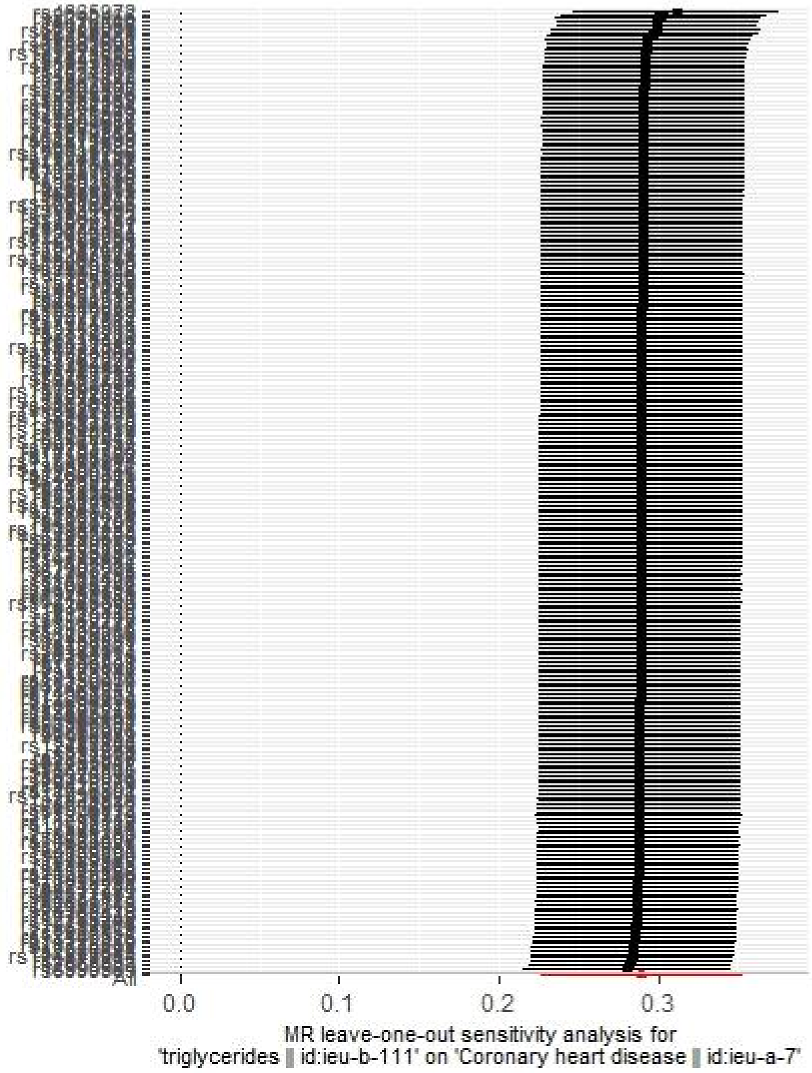

Supplemental Figure 47. Serum TG and Coronary Heart Disease TSMR leave-one-out analysis. LDL-c, low-density lipoprotein cholesterol; TSMR, two-sample Mendelian randomization

TG & CHD funnel plot

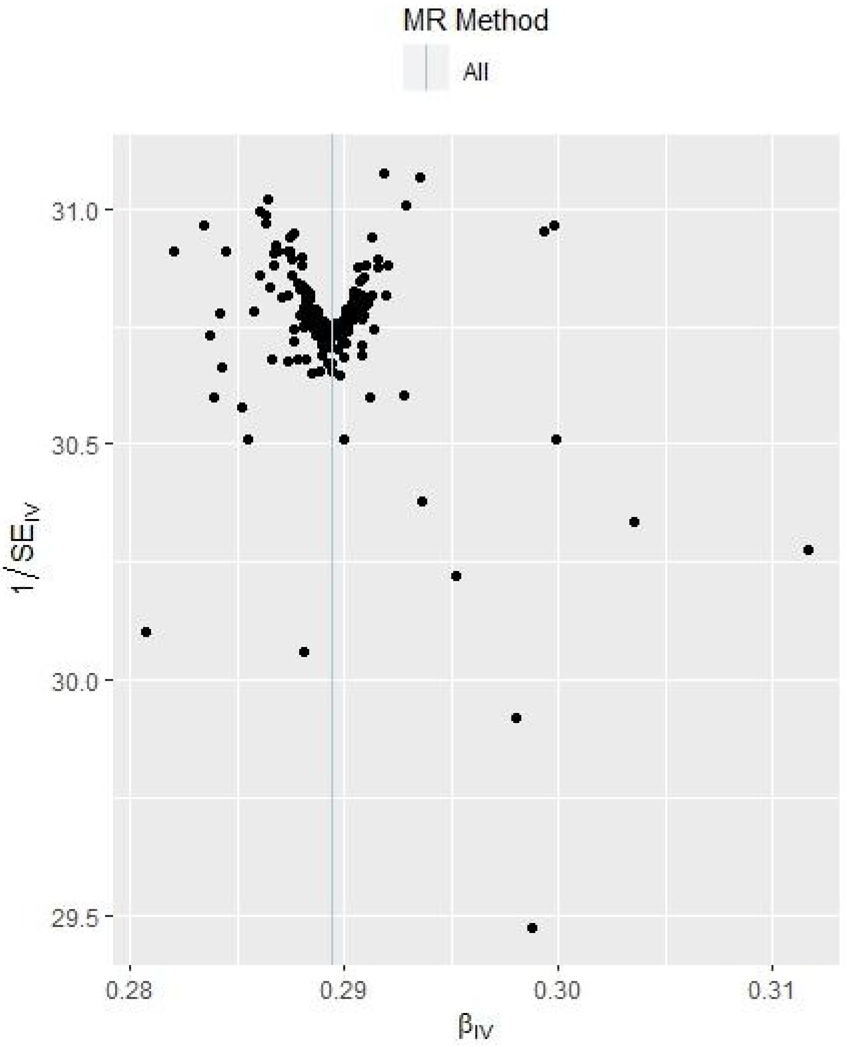

Supplemental Figure 48. Funnel plot of between serum TG and Coronary Heart Disease TSMR analysis. LDL-c, low-density lipoprotein cholesterol; TSMR, two-sample Mendelian randomization.

TC & CHD scatter plot

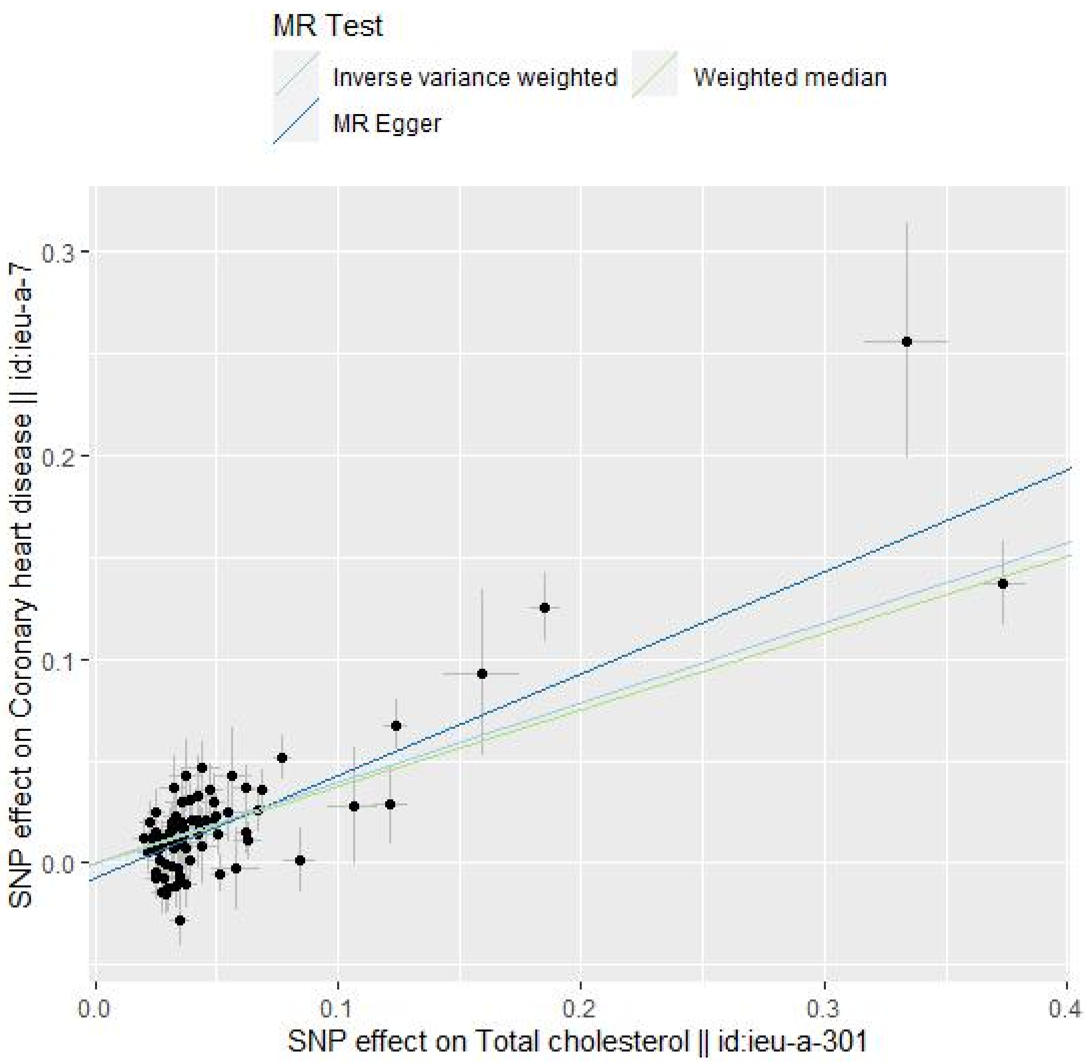

Supplemental Figure 49. Scatter plot of between serum TC and Coronary Heart Disease TSMR analysis. LDL-c, low-density lipoprotein cholesterol; TSMR, two-sample Mendelian randomization.

TC & CHD leave-one-out analysis

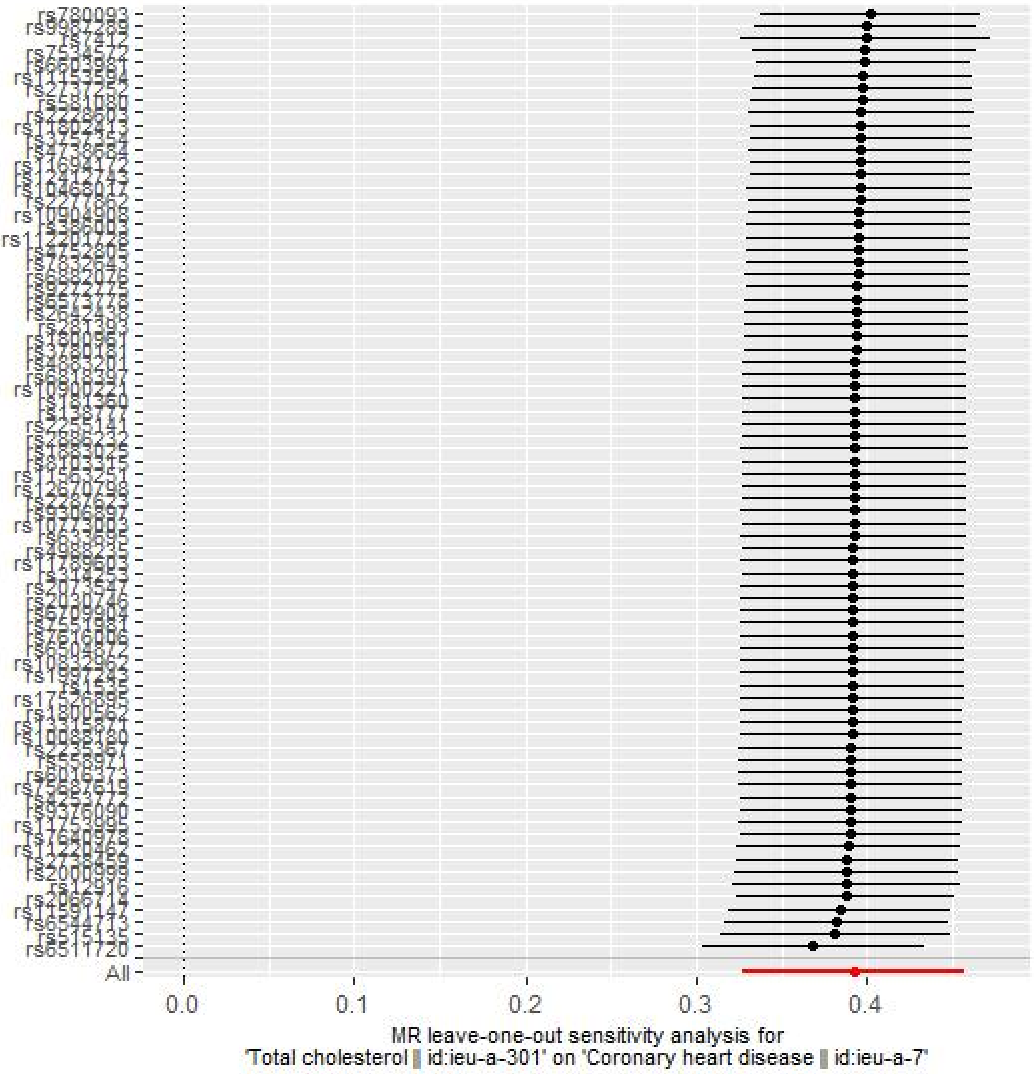

Supplemental Figure 50. Serum TC and Coronary Heart Disease TSMR leave-one-out analysis. LDL-c, lowdensity lipoprotein cholesterol; TSMR, two-sample Mendelian randomization

TC & CHD funnel plot

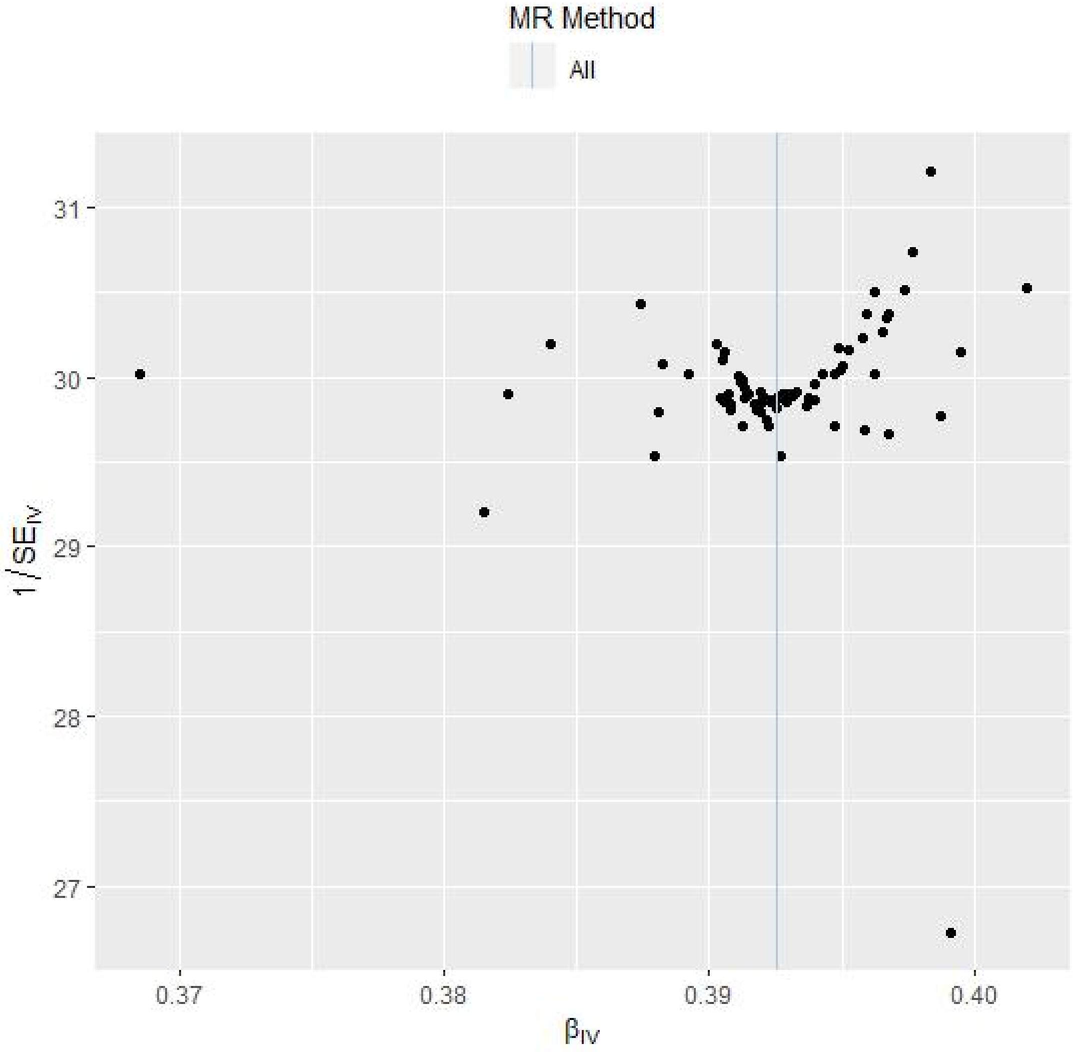

Supplemental Figure 51. Funnel plot of between serum TC and Coronary Heart Disease TSMR analysis. LDL-c, low-density lipoprotein cholesterol; TSMR, two-sample Mendelian randomization.
